# Quantum Zeno effect in the spatial evolution of a single atom

**DOI:** 10.1038/s41467-026-74332-1

**Published:** 2026-06-12

**Authors:** Zheng-Yuan Zhang, Han-Chao Chen, Xin Liu, Li-Hua Zhang, Bang Liu, Shi-Yao Shao, Jun Zhang, Qi-Feng Wang, Qing Li, Yu Ma, Tian-Yu Han, Ya-Jun Wang, Dong-Yang Zhu, Jia-Dou Nan, Yi-Ming Yin, Qiao-Qiao Fang, Dong-Sheng Ding, Bao-Sen Shi

**Affiliations:** 1 Laboratory of Quantum Information, University of Science and Technology of China, Hefei, China; 2 Synergetic Innovation Center of Quantum Information and Quantum Physics, University of Science and Technology of China, Hefei, Anhui China

**Keywords:** Matter waves and particle beams, Ultracold gases

## Abstract

The quantum Zeno effect (QZE) reveals that frequent measurements can suppress quantum evolution; however, the impact of measurements on the real-space motion of a single atom remains insufficiently explored experimentally. In this work, we employ an optical trap as a measurement pulse and, by monitoring atomic loss, directly observe the QZE in the real-space motion of a single atom. We find that the action of measurement on the atom consists of a projective measurement followed by subsequent periodic unitary evolution, thereby providing an intuitive physical picture of measurement backaction across different timescales. We further investigate the effects of measurement frequency, strength, and spatial position, demonstrating that measurements pulse not only suppress the spatial spreading of the quantum state but also enable deterministic preparation of distinct motional states. Moreover, by dynamically controlling the trap position, we realize measurement-induced directional transport of a single atom, with a velocity exceeding the maximum allowed by the adiabatic condition. Overall, our results provide a direct experimental demonstration of the QZE in real space and establish a versatile framework for measurement-based control of atomic motion, opening new possibilities for motional-state engineering in cold-atom systems.

## Abstract (web summary)

Despite substantial literature on the quantum Zeno effect, some issues such as the role of finite measurement durations are not well understood. Here, the authors demonstrate quantum Zeno dynamics in the motional states of a single trapped atom, advancing measurement-based control schemes.

## Introduction

The quantum Zeno effect (QZE)^1–5^ refers to the inhibition of coherent evolution in a quantum system through frequent measurements^6–8^, effectively freezing the quantum state by repeatedly projecting it onto a given subspace^9–11^. Originally proposed within the framework of ideal projective measurements, the concept was later generalized to scenarios involving weak measurements^12–15^ and continuous monitoring^16,17^, thereby establishing a profound connection between measurement, decoherence, and quantum state evolution^18,19^. The QZE was first predicted within the idealized framework of instantaneous orthogonal projections^1^. Later its theoretical description has been extended to a much broader scope, including continuous measurements, non-ideal weak monitoring, and non-Hermitian formulations, in all of which the QZE emerges naturally. Building on these developments, the von Neumann measurement model^20^ explicitly incorporates the measuring apparatus as a quantum degree of freedom, describing the influence of frequent measurements on system dynamics through the interaction between the system and a probe. This approach provides a unified microscopic description of measurement processes and their backaction, and it naturally accounts for the complementary anti-Zeno effect^21–23^, yielding a comprehensive theory that connects measurement frequency, measurement strength, and the intrinsic evolution of the system.

Experimentally, the QZE has been confirmed on a variety of physical platforms^2,6,8,10,24–26^. In trapped-ion systems, frequent resonant optical probing has been used to suppress coherent internal-state transitions, constituting the first direct observation of the QZE^2^. In superconducting qubit systems, continuous weak measurements via microwave cavities have enabled controllable switching between the QZE and anti-Zeno regimes^27^. Furthermore, suppression of internal-state transitions has been observed in ultracold atomic gases^28–30^ and photonic systems^31,32^. These experiments, conducted in finite-dimensional Hilbert spaces, not only confirm the central predictions of QZE theory but also demonstrate how measurement-induced constraints can be employed to engineer effective Hamiltonians.

In contrast, experimental realizations of the QZE in continuous real space, where measurements directly probe the external motional degrees of freedom^33–36^, remain relatively limited because of the stringent requirements on measurement frequency, strength, and spatial resolution. Nevertheless, some progress has been made in systems with external confinement. For example, in optical lattices, repeated high-resolution in-situ imaging of ultracold atoms has been used to suppress tunneling and diffusion within the lattice^37^. However, for particles evolving in free space, where the free time evolution of the wave function directly competes with measurement backaction, systematic experimental investigations of the QZE remain largely unexplored. It is also worth emphasizing that measurements in experiments typically have a finite duration rather than being idealized instantaneous projections. In such cases, the system becomes effectively coupled to the detector, undergoing unavoidable evolution during the measurement process, which modifies the simple freezing effect expected from frequent projections. When the measurement duration is non-negligible, the influence on system at different time scales is still insufficiently understood and calls for further investigation.

In this work, we perform a systematic experimental investigation of the influence of measurement pulses on the free-space evolution of a single atom and construct a corresponding fully quantum theoretical description. In the experiment, an atom initially confined in a trapping potential is released into the vacuum, after which a sequence of measurements is implemented through the frequent switching of the trap to influence the atomic evolution. After the atom has undergone the complete experimental sequence, in-situ imaging is performed at the trap position to determine whether the atom is lost. By repeating the experiment and statistically analyzing the atom-loss probability, we obtain a direct characterization of the atomic dynamics in real space. We first measure the dependence of the atomic motion on the measurement frequency and compare the results with numerical simulations based on a Monte Carlo approach. By continuously tuning the activation duration of the optical trap, we systematically study the backaction induced by finite-duration trapping on the system’s evolution, thereby providing a clear physical picture of backaction effects across different time scales. In addition, we demonstrate that the cooperative influence of measurement frequency, measurement strength, and measurement position enables control over the atomic motional states and can even induce directional atomic transport by varying the measurement position.

## Results

### Physical model of measurement

**Fig. 1:**
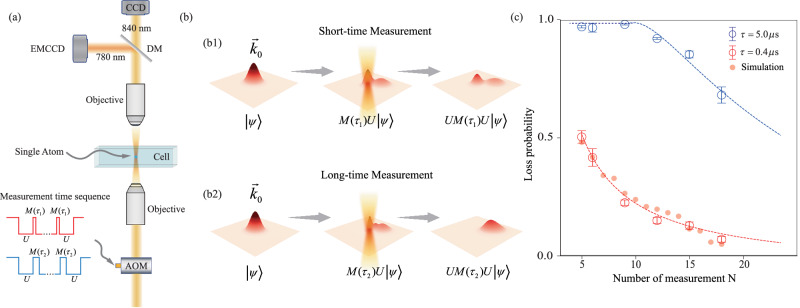
Physical diagram and observation of the Quantum Zeno Effect. **a** Experimental setup. An 840 nm laser, controlled by an acousto-optic modulator (AOM), is tightly focused by a high-NA objective into a vacuum cell to form an optical trap for a single atom from a magneto-optical trap. The time sequence applied to the AOM controls the switching of the optical dipole trap, enabling the measurement of the atom. Atomic fluorescence at 780 nm is collected by another objective and separated via a dichroic mirror. Two examples of modulation signals for short and long measurement pulses are shown on the left of the AOM. **b** Long- and short-time measurement. Top: A short pulse projects the motional state onto a trap eigenstate, leading to partial localization. Bottom: A long pulse projects the atom onto a trap eigenstate, followed by in-trap dynamics. **c** Observation of the QZE. Multiple short or long measurement pulses are applied during a 45 μs free evolution, with the measurement durations excluded from the evolution time. For the short-pulse case, the atomic loss probability decreases monotonically with increasing measurement number. The experimental data, shown as red open circles with error bars, are in good agreement with the results of Monte Carlo simulations (light-red solid points). The data are well fitted by the probability function P_loss_ = 2.92/N − 0.07 as indicated by the red dashed line. For long pulses (blue circles), the dynamics in dipole trap give rise to a clear difference compared to the short-pulse case. The decreasing scaling for measurement number *N* > 10 is fitted by P_loss_ = − 136.67(1/N−0.1)^2^ + 0.99. The error bars represent the standard deviation.

Our physical model is based on the experimental sequence illustrated in Fig. 1a. In the experiment, an 840-nm laser beam is modulated by an acousto-optic modulator (AOM) and tightly focused through a high-numerical-aperture objective to form an optical tweezer trap in the magneto-optical trap (MOT) region, which is used to capture a single cold atom and serves as an optical dipole potential^38,39^. Once a single atom is successfully trapped, an experimental run is initiated.

The atom is first released from the optical tweezer and enters the vacuum chamber, where it undergoes a period of unitary evolution in free space governed by the free-particle Hamiltonian *H*_free_ = *P*^2^/2*m*. Subsequently, a Gaussian-shaped trapping potential is switched on again and maintained for a finite duration *τ*. Physically, the switching-on of the trap contains two inseparable ingredients. The first is a measurement process that determines whether the atomic wavefunction still lies within the bound-state subspace of the trapping potential. The second is the subsequent unitary evolution under the trap Hamiltonian:

1 $${H}_{{{\rm{trap}}}}={H}_{{{\rm{free}}}}-{U}_{0}\exp \left(-\frac{2{r}^{2}}{{\omega }_{0}}\right),$$

where *U*_0_ denotes the trap depth, *r* is the distance of the atom from the trap center, and *ω*_0_ is the beam waist of the optical tweezer. If the atom is not lost during this measurement process, its wavefunction continues to participate in the subsequent dynamics. After the trap is switched off again, the atom is released and enters the next experimental cycle. After completing the full sequence of measurements, the atomic position is detected via fluorescence imaging at the trap location, which determines whether the atom ultimately remains trapped.

Based on the physical picture described above, we construct a theoretical model in which the atomic dynamics are described by a repeated four-stage sequence: release from the trap, free evolution, measurement, and subsequent in-trap evolution. Using exactly the same parameters as in the experiment, we then perform quantitative Monte Carlo simulations of the atomic dynamics. In the experiment, a single atom is initially released from a Gaussian optical trap with depth *U*_0_ = 2mK and beam waist *ω* = 1.2 μm. Taking into account the finite atomic temperature *T*_atom_ = 175 μK ^40^ in the trap, the initial state $| {\psi }_{0}\rangle$ is described as a thermal mixed state obeying the corresponding statistical distribution. In the simulations, the $| {\psi }_{0}\rangle$ is generated by randomly sampling all bound states of the trapping potential according to the Boltzmann distribution. Specifically, the probability for the atom to occupy the *n*-th bound state $| {{{\rm{b}}}}_{n}\rangle$ of the trapping Hamiltonian *H*_trap_ is given by:

2 $${{{\rm{P}}}}_{n}\propto \exp \left(-\frac{{E}_{n}}{{k}_{{{\rm{B}}}}{T}_{{{\rm{atom}}}}}\right),$$

where *E*_*n*_ is the eigenenergy of $| {{{\rm{b}}}}_{n}\rangle$, and *k*_B_ is the Boltzmann constant.

After release, the atom undergoes free-space evolution *U* for a duration *t*, after which the optical tweezer trap is switched on again. At this moment, the atomic wavefunction $| {\psi }_{t}\rangle$ is subjected to a projective measurement in the basis of all bound eigenstates $\{| {b}_{i}\rangle \}$ of the trap. The atom collapses into a specific bound state with probability ${{{\rm{p}}}}_{n}=| \langle {{{\rm{b}}}}_{n}| {\psi }_{t}\rangle {| }^{2}$, while the total probability that the atom is not captured by any bound state is defined as the loss probability during this measurement, denoted by ${{{\rm{p}}}}_{{{\rm{loss}}}}=1-{\sum }_{n}| \langle {{{\rm{b}}}}_{n}| {\psi }_{t}\rangle {| }^{2}$. In the numerical simulations, the measurement back-action is implemented through a stochastic sampling procedure, where the outcome of each measurement is determined by random number generation. If the atom survives the measurement, the updated wavefunction subsequently evolves under the trap Hamiltonian for a duration *τ*, after which the system enters the next cycle.

After completing the full sequence of measurements, the experiment determines whether the atom remains trapped via fluorescence imaging. Correspondingly, in the numerical simulations, the outcome of each stochastic process determines whether the atom ultimately remains in the trap. By repeating the above procedure for a sufficiently large number (*N*_total_) of independent realizations, we count the number of surviving events *N*_surv_, from which the atomic loss probability P_loss_ = 1 − *N*_surv_/*N*_total_ is extracted. Experimentally, each data set consists of 500 repeated experimental runs, and each data point is obtained by averaging over three independent data sets under identical parameters. Due to the probabilistic loading of single atoms from the MOT, the effective number of realizations per data set is ~300. In the Monte Carlo simulations, we directly use 500 trajectories.

This model not only provides an intuitive description of the measurement-induced non-unitary collapse of the wavefunction, but also naturally incorporates the unitary dynamics of the atom in both free space and the trapping potential within a unified theoretical framework.

### Quantum Zeno effect of a single atom

In the experiment, the total free-space evolution time of the atom is fixed at T = 45 μs. During this evolution, N pulse-shaped trapping potentials of duration *τ*, each containing a measurement process (hereafter referred to as measurement pulses), are inserted at equal time intervals. This protocol allows us to systematically investigate the influence of finite-duration pulses on the atomic dynamics. We focus on two representative cases: short measurement pulses dominated by the measurement process (*τ*_1_ = 0.4 μs) and long measurement pulses that involve pronounced unitary evolution inside the trap (*τ*_2_ = 5.0 μs).

In the short-pulse regime, the action of a measurement pulse on the atomic motional state, denoted as *M*(*τ*_1_), can be well approximated by an ideal projective measurement onto the bound-state subspace, ${M}_{{{\rm{b}}}}={\sum }_{n}| {{{\rm{b}}}}_{n}\rangle \langle {{{\rm{b}}}}_{n}|$. Under this action, the atomic wavefunction becomes primarily localized within the spatial region covered by the Gaussian trapping potential after the pulse, with only minor leakage along the direction of the initial group velocity. Once the trap is switched off, the wavefunction continues to expand into free space, as illustrated in Fig. 1b1. Considering that, during a single free-evolution interval of duration T/N, the atomic loss probability is given by:

3 $$\mathop{\sum }\limits_{n}| \langle {{{\rm{b}}}}_{n}| {H}_{{{\rm{free}}}}| {\psi }_{0}\rangle {| }^{2}\times {{{\rm{T}}}}^{2}/({\hslash }^{2}{{{\rm{N}}}}^{2}),$$

the cumulative loss probability after N measurements is therefore proportional to 1/N (see Methods for details). The corresponding experimental results are shown by the red data points with error bars in Fig. 1c: as the number of measurements increases, the loss probability rapidly approaches zero following an inverse dependence, indicating that frequent projective measurements effectively freeze the spatial evolution of a single atom, giving rise to the QZE. Monte Carlo simulations of the same process, shown as lighter red dots, exhibit good agreement with the experimental trend.

In contrast, in the long-pulse regime, the wavefunction undergoes substantial unitary evolution inside the trap following the collapse induced by the measurement (Fig. 1b2). In this case, the action of a measurement pulse on the atomic motional state can be expressed as:

4 $$M({\tau }_{2})={e}^{-i{H}_{{{\rm{trap}}}}{\tau }_{2}/\hslash }{M}_{{{\rm{b}}}}$$

which leads to a larger fraction of the wavefunction components extending beyond the trapping region at the end of a single pulse. This behavior arises because, during the evolution inside the trap, the atomic wavefunction acquires an additional phase distribution, causing its subsequent free-space evolution to depend sensitively on the residence time within the trap. This effect is reflected in the blue curve in Fig. 1c. For *τ* = 5.0 μs, the duration of the measurement pulse corresponds to one half of the trap oscillation period. In an idealized picture, the unitary evolution of the atomic wavefunction inside the trap drives its spatial distribution back toward a localized state near the trap center at the end of the pulse, such that the atomic loss probability remains at a relatively low level (see the next section). However, the above discussion primarily addresses atom loss arising from the reduced overlap between the atomic wavefunction and the bound-state subspace. In the actual experiment, coupling between the atom and the environment introduces additional loss channels. The increased total evolution time associated with long pulses, therefore, leads to a noticeably higher loss probability at low measurement frequencies compared with the short-pulse case. As the measurement frequency is increased, the measurement-induced quantum Zeno effect gradually becomes dominant, and the atomic loss probability correspondingly decreases with increasing measurement frequency.

It should be further noted that both excessively high and excessively low temperatures hinder the clear observation of the quantum Zeno effect in the experiment. Since the single atoms confined in the optical tweezer are directly loaded from a magneto-optical trap, environmental noise increases with atomic temperature, enhancing the probability of atom loss from the trap. As a result, at higher temperatures, the atomic loss probability after the full experimental sequence approaches unity regardless of the measurement pulse frequency, thereby obscuring the characteristic signatures of the quantum Zeno effect. Conversely, at low temperatures, the intrinsic atom-loss probability within a single release window is already close to zero, making the additional suppression induced by increasing the measurement frequency difficult to resolve against statistical noise.

### Measurement-induced periodic dynamics

**Fig. 2:**
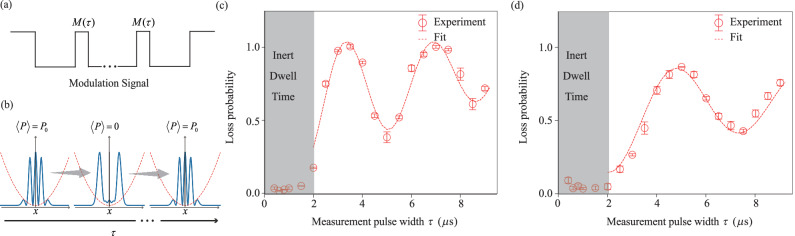
Dynamics of a single atom under finite-time measurements. **a** Measurement time sequence used in the experiment. The total free evolution time is 30 μs, with *N* = 15 inserted measurements. The pulse duration is varied to probe the motional dynamics in the trap. **b** Schematic of atomic dynamics in a Gaussian trap. The atomic wave packet undergoes periodic oscillations under the confinement of the trap: it is initially localized near the trap center with an initial momentum *P*_0_; after one quarter of the trap oscillation period, the momentum reduces to *P* = 0, and the spatial spread of the wave packet relative to the trap center reaches its maximum; after another quarter period, the wave packet evolves back to a distribution close to its initial state and subsequently follows a dynamical evolution symmetric to that of the first half-cycle. **c** Atom loss probability versus measurement pulse width. For pulse widths below  ~ 2.0 μs, the loss probability remains nearly zero, corresponding to the Inert-Dwell-Time regime. For longer pulses, the loss probability exhibits periodic oscillations, reflecting in-trap dynamics. The red dashed curve is a damped oscillatory fit ${{{\rm{P}}}}_{{{\rm{loss}}}}=0.5{e}^{-0.11\tau }\sin (1.77\tau+1.94)+(1-0.5{e}^{-0.11\tau })$. **d** Atom loss with reduced pulse intensity (half of (**c**)). The Inert-Dwell-Time regime remains  ~ 2.0 μs, while the oscillation period increases as the trap depth decreases, fitted by ${{{\rm{P}}}}_{{{\rm{loss}}}}=0.415{e}^{-0.09\tau }\sin (1.19\tau+2.12)+0.83(1-0.5{e}^{-0.11\tau })$. The error bars represent the standard deviation.

To explore the influence of measurement pulses on atomic motion across different timescales, we continuously vary the measurement pulse width and quantitatively characterize the atomic dynamics by monitoring the loss rate. The experimental sequence is shown in Fig. 2a: *N* = 15 measurement pulses of duration *τ* are inserted into a 30*μ*s free-evolution window. Under the action of the measurement pulses, the atomic wavefunction is projected into the bound-state manifold and subsequently undergoes periodic oscillations driven by the trapping potential. Within one full oscillation period, the evolution of the wavefunction in the first half-cycle is symmetric to that in the second half-cycle, and the atomic momentum magnitude reaches zero and its maximum value twice during each period. A schematic illustration of this process is shown in Fig. 2b. Owing to this symmetry in the atomic motion within the trap, when the measurement pulse duration is scanned from 0 to one full oscillation period, the atomic loss probability exhibits two symmetric peaks. Correspondingly, the oscillation frequency of the loss probability as a function of the measurement pulse duration is twice the intrinsic oscillation frequency of the trapping potential.

The experimental results corresponding to the above process are shown in Fig. 2c. When the measurement pulse duration is shorter than  ~2.0 *μ*s, the atom exhibits negligible loss throughout the experiment. This characteristic timescale is independent of the trap parameters but decreases with longer free-evolution windows between trap switch-off events. At the critical value, the trap-induced spatial displacement of the wavefunction, combined with the subsequent free-space expansion, is just sufficient to drive the atom beyond the trapping region. By contrast, within this threshold, the trap-induced displacement remains too small for the atom to escape. We refer to this timescale as the Inert Dwell Time.

Once the measurement pulse duration exceeds this threshold, the loss probability exhibits damped oscillations as a function of the measurement duration. Considering a trap depth of *U*_0_ = 2mK and a beam waist of *ω*_0_ = 1.2*μ*m, the oscillation period of a ^87^Rb atom confined in the trap is approximately $\sqrt{{m}_{{{\rm{Rb}}}}{\omega }_{0}^{2}/(4{U}_{0})}\approx 8.3\,\mu {{\rm{s}}}$, where *m*_Rb_ denotes the atomic mass^40,41^. This period is approximately twice the oscillation period observed in the experimental loss probability as a function of *τ*, in good agreement with the theoretical expectation, indicating that the loss probability faithfully reflects the periodic motion of the atom inside the trap. The damping observed in the data originates from dissipative coupling between the atom and its thermal environment, as well as from higher-order axial modes of the trapping potential, both of which effectively modulate the oscillation amplitude. A sinusoidal function with an exponential envelope provides a good fit to the experimental data. Furthermore, by reducing the measurement pulse intensity by half, we tune the effective trap frequency. As shown in Fig. 2d, the Inert Dwell Time ( ~ 2.0 μs) remains unchanged, while the oscillation period of the loss probability doubles, demonstrating that the back-action of long measurement pulses on the atom is indeed dominated by its unitary evolution inside the trap.

These results indicate that finite-duration measurements applied to a single atom are governed by distinct physical mechanisms on different timescales: Short pulses act as near-projective probes, collapsing the atomic wavefunction into localized eigenstates, whereas long pulses induce coherent in-trap oscillations that lead to quantitative displacements of the wavefunction. Such timescale-dependent control indicates the potential of measurement as a powerful resource for preparing and manipulating motional quantum states^42–45^.

### Crossover of measurement strength

The measurement strength plays a central role in determining the dynamical response of a quantum system. In general, sufficiently weak measurements impose only limited perturbations on the motional state, whereas strong measurements enforce wavefunction collapse through orthogonal projection. To investigate how trap-based measurements of different strengths influence the motion of a single atom, we tune the measurement strength by varying the intensity of the measurement pulses and monitor the resulting motional dynamics during free evolution.

**Fig. 3:**
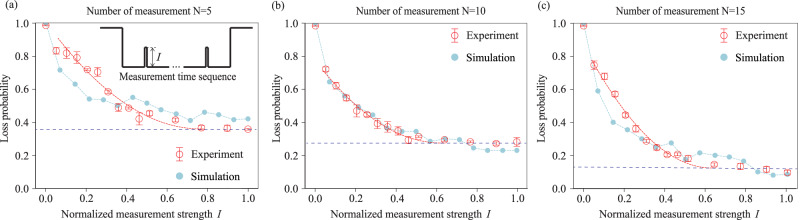
Evolution under different measurement strengths. During a 30 μs free evolution, N equally spaced measurement pulses of 0.4 *μ*s duration are inserted. **a**–**c** show the final atom loss probability as a function of measurement strength for *N* = 5, 10, 15, respectively. The experimental data are represented by red open circles, while the simulated results are indicated by light-blue filled circles. The inset in (**a**) displays measurement time sequence used in the experiment, where the level *I* means the measurement strength. For normalized strength below  ~ 0.6, the loss probability decreases markedly with increasing strength, while above  ~ 0.6 the decrease becomes much slower, thus defining the strong measurement regime. The decreasing scaling is fitted by a quadratic function P_loss_ = 1.10(*I*−0.77)^2^ + 0.36 (*N* = 5), P_loss_ = 1.28(*I*−0.63)^2^ + 0.26 (*N* = 10), P_loss_ = 1.87(*I*−0.63)^2^ + 0.13 (*N* = 15), respectively. The error bars represent the standard deviation.

The measured atom-loss probabilities as a function of measurement strength are shown in Fig. 3a-c. The inset of Fig. 3a illustrates the experimental time sequence: N measurement pulses of 0.4 μs duration are embedded within a 30 μs free-evolution window. On this timescale, the unitary evolution induced by the trapping potential is essentially negligible. The variation of the normalized measurement strength *I* is implemented by adjusting the trap depth, with the reference depth set to 2mK. In the weak-measurement regime, each measurement pulse perturbs the atomic motional state, effectively reducing the outward propagation rate of the wavefunction and thereby slightly suppressing the loss probability compared to free evolution. As the measurement pulse intensity is gradually increased, the influence of a single measurement on the spatial wavefunction becomes progressively stronger. This suppression exhibits a quadratic scaling with measurement strength until *I* exceeds a threshold value (~0.6), beyond which a single measurement can be well approximated as an ideal projective measurement. In this regime, the recapture probability during an individual measurement is primarily determined by the initial atomic temperature and the evolution time within the release window. Further increasing the measurement strength does not significantly modify the projection induced by each pulse; consequently, the atom-loss probability accumulated over multiple measurements approaches a limiting value determined by the measurement frequency, as indicated by the black dashed line in the figure. Under conditions of frequent strong measurements [Fig. 3c], the atom-loss probability approaches zero, corresponding to a freezing of the motional dynamics induced by the quantum Zeno effect. These results clearly reveal a crossover from weak-measurement-induced motional perturbations to strong-measurement-dominated wavefunction collapse, and demonstrate that the combined control of measurement strength and measurement frequency provides a flexible and powerful approach for engineering atomic motional quantum states^46,47^.

### Manipulation of motional state

**Fig. 4:**
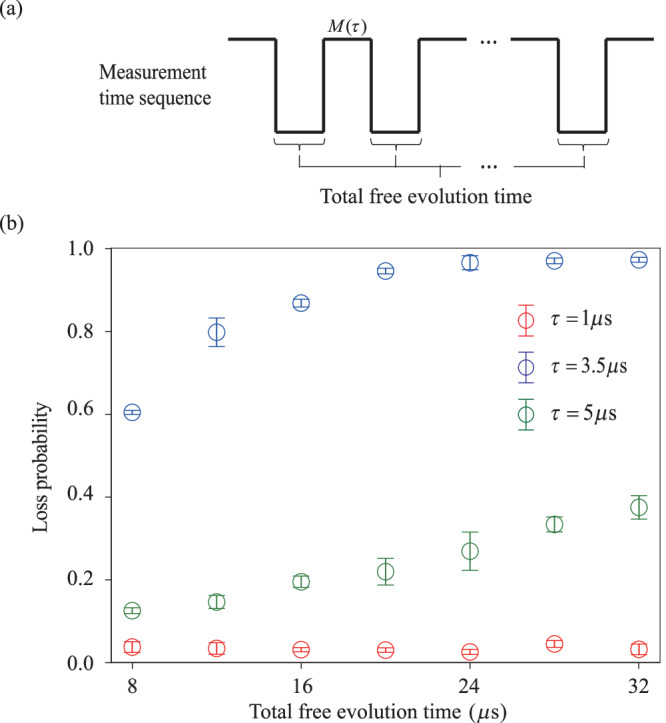
Free evolution interrupted by repeated measurements. **a** Experimental sequence. During free evolution, measurement pulses are inserted every 2 μs, with pulse widths set to 1 μs, 3.5 μs, and 5 μs, respectively. **b** Atom loss probability as a function of the total free evolution time for different pulse durations. For 1 μs pulses (red open circles), the loss probability remains negligible and independent of the evolution time. For 3.5 μs pulses (blue open circles), the loss probability increases rapidly with evolution time and approaches nearly unity beyond 24 μs. In contrast, for 5 μs pulses (green open circles), the loss probability rises much more slowly and stays below 0.4 even at 32 μs, indicating a substantial suppression of atom loss compared with the 3.5 μs case. The error bars represent the standard deviation.

Building on the above results, we combine control over the duration, strength, and frequency of the measurement pulses with adjustment of the total free-evolution window to demonstrate directional control of the atomic motional state. The experimental sequence is shown in Fig. 4a: during each free-evolution interval, measurement pulses are applied every 2 μs. By extending the total free-evolution time while keeping the pulse spacing fixed, we monitor the motion of atom under different measurement conditions via the loss probability.

When the goal is to suppress the atomic motion, frequent short measurement pulses (τ = 1 μs) strongly localize the atomic wave packet near the center of the trapping potential, allowing it to remain in a low-loss motional state even during long intervals of free evolution [Fig. 4b, red data points]. In contrast, when the objective is to facilitate the escape of atoms from the trap, we apply measurement pulses with a duration matched to one quarter of the trap oscillation period, τ = 3.5 μs. In this regime, the unitary evolution inside the trap induces an effective phase inversion of wavefunction components that would otherwise favor confinement, thereby degrading their spatial localization during the subsequent free evolution and significantly reducing the probability of atomic recapture [Fig. 4b, blue data points].

Notably, although thermal noise introduces additional loss channels for atoms inside the trap, the atomic loss probability does not vary monotonically with the measurement pulse duration. Specifically, when the pulse duration is extended to half of the trap oscillation period, τ = 5.0 μs, the oscillatory dynamics of the trap partially restore the spatial localization of the wave packet, leading to a reduced loss probability during the release process. Experimentally, this manifests as the reemergence of low-loss behavior at specific evolution times, as indicated by the green data points in Fig. 4b. These results clearly demonstrate that measurements can be employed not only to suppress or enhance atomic motion but also, when properly engineered, to serve as a versatile resource for tailoring and controlling atomic motional states, enabling effective manipulation of atomic dynamics^48,49^.

### Directional atomic motion

**Fig. 5:**
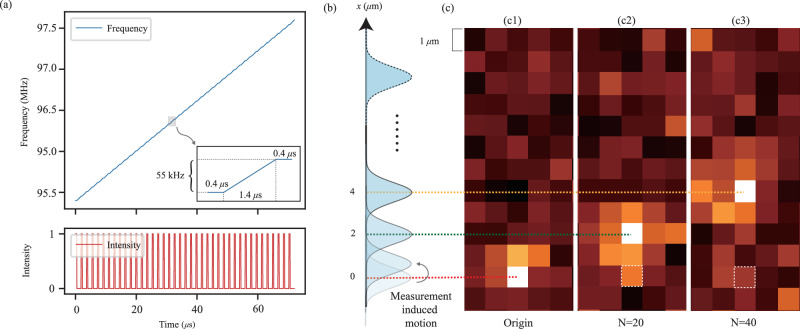
Controlled atomic motion via frequent measurements. **a** Measurement time sequence. The upper panel shows the frequency control sequence of the acousto-optic deflector (AOD) driving signal, with the frequency increasing stepwise from 95.4MHz. The lower panel shows the intensity control sequence of the AOD driving signal, corresponding to the timing of the applied measurements. The measurement pulse width is 0.4 μs, with a 1.4 μs interval between measurements. The inset in the upper panel shows that the frequency variation of 55kHz for every step occurs within the 1.4 μs interval when the intensity is zero. **b** Schematic of atomic wave packet motion. Each measurement pulse induces a slight shift in the center position relative to the previous one, resulting in a corresponding displacement of the atomic wave packet’s center after projection. After multiple such measurements, the center of the atomic wave packet undergoes a noticeable shift. **c** Experimental results. **c1** Atomic position imaging before any measurements are applied. **c2** Atomic position imaging after *N* = 20 measurements, showing a displacement of approximately 2 μm from the initial position. **c3** Atomic position imaging after *N* = 40 measurements, showing a displacement of ~4 μm from the initial position. Both displacements are measured relative to the position before measurements were applied.

Building upon in-situ measurements of the atom, we further introduce a series of projective measurements with gradually shifted spatial positions. Experimentally, this scheme can be realized by replacing the AOM in the optical path with an acousto-optic deflector (AOD). By jointly controlling the frequency and intensity of the radio-frequency (RF) signal applied to the AOD, each measurement is displaced by a fixed offset relative to the previous one. The control sequence is illustrated in Fig. 5a, where the inset depicts a single step of the process: after applying a 0.4 μs projective strong measurement, the dipole trap is switched off (corresponding to zero RF intensity), followed by 1.4 μs of free-space evolution. During this free evolution window, a frequency shift is applied to the RF drive. At the end of this interval, a signal with the same intensity but a frequency 55kHz higher than the previous one is applied to the AOD. In our experimental system, this control sequence results in each measurement trap being displaced by ~0.1 μm relative to the preceding one. Repeating this process N times, the measurement backaction on the atom can be expressed as $M={({\prod }_{j=0}^{N}{M}_{j})}^{{\dagger} }$, where ${M}_{j}={\sum }_{n}| {{{\rm{b}}}}_{n}^{j}\rangle \langle {{{\rm{b}}}}_{n}^{j}|$ denotes the measurement operator corresponding to a position shifted by *j* units of *Δr*_0_ relative to the origin, that is, the trapping potential is turned on at a distance *jΔr*_0_ from the origin.

Under such a sequence, the role of frequent measurements is no longer to freeze the atom at a fixed position. Instead, the atomic wavefunction is repeatedly projected in a directional manner, with the wave packet center being transferred step by step to the position of the dipole trap, i.e., measurement-induced directed motion of the atom, as illustrated in Fig. 5b. Experimentally, we first perform in-situ imaging of the atom (Fig. 5c1), followed by global imaging for measurement numbers *N* = 20 (Fig. 5c2) and *N* = 40 (Fig. 5c3). The results clearly show the atom bound in the dipole trap displaced by ~2 μm and 4 μm from the initial position, respectively. It is worth emphasizing that, in this process, the effective drift velocity of the atom is ~0.7m/s, which is significantly higher than the maximum velocity of 0.05m/s achievable through trap dragging with the optical tweezer kept continuously on in the same experimental system. This result demonstrates that measurement-induced directed atomic motion is no longer constrained by the adiabatic condition associated with unitary dynamics, thereby providing a new paradigm for controlling atomic displacement^50^.

From a fundamental perspective, however, the maximum transport rate achievable with this measurement-induced scheme is still limited by the atomic temperature. Higher temperatures lead to more rapid expansion of the atomic wavefunction during free evolution, and thus, for a given time interval, require a corresponding reduction in the spatial separation between successive measurement positions in order to maintain sufficient overlap with the bound states of the trap. Otherwise, atom loss induced during the measurement process can substantially reduce the efficiency of atomic control. Consequently, this approach is particularly well suited for applications involving low-temperature atoms and scenarios in which a certain degree of tolerance to single-step loss is acceptable.

## Discussion

In this work, we employ short opening pulses of an optical trap as a means of measurement control to systematically investigate the influence of measurement pulses on the evolution of a single cold atom in free space. By comparing the dynamical effects induced by frequent measurement pulses with different durations, we directly observe, in real space, the freezing of atomic dynamics under high-frequency measurements, namely the QZE, and clearly reveal the fundamental differences in atomic dynamics governed by projection-measurement-dominated and unitary-evolution-dominated mechanisms. Furthermore, we find that as the duration of the measurement pulses increases, the atomic dynamics exhibit a distinctive structure: an initial nearly frozen stage followed by periodic oscillatory motion. The oscillation period is determined by the strength of the Gaussian trap, whereas the duration of the frozen stage is insensitive to the trap strength and can therefore be approximately regarded as an instantaneous projective measurement. This result provides a clear physical picture for understanding finite-duration measurements, indicating that a realistic measurement is not an idealized instantaneous projection but rather a process composed of multiple dynamical stages on different time scales.

Building on this, we further systematically examine the combined influence of measurement frequency and measurement strength on the free evolution of a single atom. Our experimental results show that measurements can not only effectively suppress or delay the spatial spreading of the quantum state, but also enable the deterministic preparation of different motional states through appropriate tuning of the measurement parameters. Beyond in-situ measurements, by precisely controlling the spatial position at which the measurements are applied, we also realize directional motion of the atomic wavefunction, thereby overcoming the limitation imposed by the adiabatic condition and achieving a significantly higher atomic transport velocity. Overall, this work provides a clear experimental demonstration of the QZE in real space for a freely evolving particle, elucidates the dynamical role of measurement in realistic experimental settings, and establishes an experimental and conceptual foundation for further exploration of measurement-induced dynamics^51–53^. These results open a feasible pathway toward motional state engineering in cold-atom systems^54–56^.

## Methods

### Quantum Zeno scaling of the atom loss probability

We consider the free evolution of a single atom over a total duration T, during which N projective measurements are inserted at equal intervals of T/N. The atom is initially prepared in the state $| {\psi }_{0}\rangle$ immediately after release from the trap. After a free evolution of duration T/N, the state evolves to ${U}_{{{\rm{free}}}}({{\rm{T}}}/{{\rm{N}}})| {\psi }_{0}\rangle={e}^{-i{H}_{{{\rm{free}}}}{{\rm{T}}}/(\hslash {{\rm{N}}})}| {\psi }_{0}\rangle$. A measurement operator ${M}_{{{\rm{b}}}}={\sum }_{n}| {{{\rm{b}}}}_{n}\rangle \langle {{{\rm{b}}}}_{n}|$ is then applied, the post-measurement state thus becomes ${\sum }_{n}\langle {{{\rm{b}}}}_{n}| {e}^{-i{H}_{{{\rm{free}}}}{{\rm{T}}}/\hslash {{\rm{N}}}}| {\psi }_{0}\rangle | {{{\rm{b}}}}_{n}\rangle$, and the atom loss probability is ${{{\rm{P}}}}_{1}=1-| {\sum }_{n}\langle {{{\rm{b}}}}_{n}| {e}^{-i{H}_{{{\rm{free}}}}{{\rm{T}}}/\hslash {{\rm{N}}}}| {\psi }_{0}\rangle {| }^{2}$.

For sufficiently small T/N, the propagator can be expanded to first order, yielding the approximation ${\sum }_{n}\langle {{{\rm{b}}}}_{n}| {e}^{-i{H}_{{{\rm{free}}}}{{\rm{T}}}/\hslash {{\rm{N}}}}| {\phi }_{0}\rangle \approx 1-i{\sum }_{n}\langle {{{\rm{b}}}}_{n}| {H}_{{{\rm{free}}}}| {\phi }_{0}\rangle {{\rm{T}}}/(\hslash {{\rm{N}}})$. This leads to the expression of the single-measurement loss probability ${{{\rm{P}}}}_{1}={\sum }_{n}| \langle {{{\rm{b}}}}_{n}| {H}_{{{\rm{free}}}}| {\psi }_{0}\rangle {| }^{2}{{{\rm{T}}}}^{2}/({\hslash }^{2}{{{\rm{N}}}}^{2})$. Extending this to N repeated measurements gives the final loss probability ${{{\rm{P}}}}_{{{\rm{N}}}}=1-{{{\rm{P}}}}_{1}^{{{\rm{N}}}}$. Taking the limit N → *∞*, we obtain the first order approximation ${{{\rm{P}}}}_{{{\rm{N}}}}={\sum }_{n}| \langle {{{\rm{b}}}}_{n}| {H}_{{{\rm{free}}}}| {\psi }_{0}\rangle {| }^{2}{{{\rm{T}}}}^{2}/({\hslash }^{2}{{\rm{N}}})$, which predicts that the atom loss probability decreases linearly with increasing measurement number.

### Magneto-optical trap loading

To stochastically load a single ^87^Rb atom into the dipole trap, a magneto-optical trap (MOT) is first established at the trap site. The MOT employs three intersecting retroreflected beams containing both cooling and repump light, together with an anti-Helmholtz magnetic field. One pair of beams is oriented perpendicular to the optical table, while the other two pairs are confined to lie within the plane of the table. Due to the short working distance ( ~ 15mm) of the high-numerical aperture objective, the in-plane beams cannot intersect at the conventional 90^∘^, but instead cross at an angle of  ~ 120^∘^. Each cooling beam has a diameter of  ~ 20mm and a power of  ~ 6mW, while each repump beam carries  ~ 1mW. The magnetic field gradient is set to  ~ 14G/cm. The cooling light is red-detuned by 15MHz from the $| 5{S}_{1/2},F=2\rangle \to | 5{P}_{3/2},F=3\rangle$ transition, and the repump light is resonant with the $| 5{S}_{1/2},F=1\rangle \to | 5{P}_{3/2},F=2\rangle$ transition.

Rubidium atoms are supplied from a thermally heated dispenser, with atomic flux regulated by a tunable valve. For single-atom trapping, the valve is adjusted to its minimum opening, thereby reducing the MOT loading rate and suppressing background collisions that would otherwise limit the trap lifetime.

### Single-atom optical tweezer

A single-atom optical trap is realized by tightly focusing an 840 nm far-off-resonant Gaussian beam using a custom high-numerical aperture (N.A. = 0.5) objective, yielding a beam waist of 1.2 μm. At this scale, collisional blockade^39^ ensures that the trap hosts at most one atom. The trapping beam power is set to 8mW, corresponding to a trap depth of 2.0mK, with a single-atom lifetime of several seconds under a vacuum level below 1 × 10^−8^Pa, limited primarily by off-resonant photon scattering and residual background gas collisions. Under the experimental conditions employed in this work, the scattering or heating rate induced by the optical tweezer is expected to be on the order of a few hertz. The trapping beam is intensity-controlled and rapidly switched using an acousto-optic modulator (AA MT80-B30A1-IR), which is driven by an arbitrary waveform generator to flexibly program the pulse sequences and intensities.

### Fluorescence detection

Trap occupancy is detected by fluorescence imaging. Two probe beams, one red-detuned by 30MHz from the cooling transition and another resonant with the repump transition, are applied perpendicular to the dipole-trap axis to minimize background scattering. Each probe beam has a diameter of  ~ 1mm; the cooling probe carries  ~ 0.4mW of power, while the repump probe carries  ~ 0.1mW. Atomic fluorescence is collected by a second objective, spectrally separated from the 840-nm light by a dichroic mirror, and recorded on an electron-multiplying CCD (EMCCD, ANDOR iXon Ultra 897) with EM Gain = 100.

To further suppress background contributions, the dipole-trap beam is spectrally filtered with three 840-nm bandpass filters before the high-NA objective, while three additional 780 nm bandpass filters are placed in front of the EMCCD to block stray light at other wavelengths. This filtering scheme is crucial for achieving a high signal-to-noise ratio, enabling unambiguous discrimination of single-atom fluorescence against residual trap light and scattered background.

### Experimental time sequence

The sequence for preparing a single atom proceeds as follows: the MOT and dipole-trap beams are switched on simultaneously. After 140ms, the MOT beams and magnetic field are turned off, allowing a single atom to be loaded in the dipole trap during the ensemble decay. Following a 25 ms wait, the probe beams and EMCCD are activated for 20 ms to record the atomic fluorescence. The 25 ms delay ensures that the residual cold atomic cloud has sufficiently dispersed, thereby preventing background atoms from contributing to the subsequent fluorescence signal used to determine single-atom loading.

Once a single atom is prepared in the trap, the experimental measurement sequence is applied. After the completion of the measurement protocol, a second fluorescence image is acquired 40ms after the initial detection. The interval between the two images is chosen to accommodate the readout time of the EMCCD, while still allowing reliable detection of the atom at the end of the experimental cycle. The first fluorescence image serves to identify successful single-atom loading, whereas the second image provides the readout of the experimental outcome.

### Single-atom discrimination

**Fig. 6:**
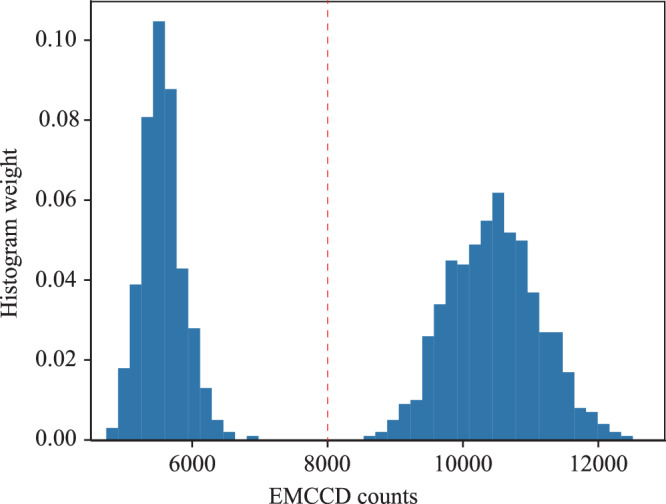
Histogram of single-atom fluorescence counts. The histogram shows the EMCCD count distribution from 1000 repeated fluorescence imaging experiments. The histogram exhibits a clear bimodal structure, with peaks centered at ~5500 and 10,500 counts, corresponding to empty traps and single-atom occupancy, respectively. The red dashed line denotes the threshold used to discriminate successful single-atom loading.

The fluorescence signal is used to discriminate single-atom occupying events. To balance signal strength with acquisition time, the fluorescence imaging duration is set to 20ms, which provides sufficient photon counts for reliable atom detection without introducing excessive delay to the experimental cycle. A histogram of photon counts from 1000 experimental realizations displays a clear bimodal distribution, corresponding to zero or one atom in the trap (Fig. 6). The threshold between the two peaks defines the single-atom loading criterion. The statistical analysis yields a stochastic loading probability of 0.5–0.6. The absence of any higher-photon-count peak confirms the collisional blockade limit, ensuring that at most one atom is present in the trap.

### Adjustable measurement pulse position control

To realize measurement pulses with adjustable center positions, the dipole trap light passes through an acousto-optic deflector (AOD, AA DTSXY-400-800) before entering the high-numerical-aperture objective. By tuning the frequency and intensity of the AOD’s driving signal, we implement the required sequence for repeated measurements in the experiment. In this setup, the frequency difference between the driving signals of consecutive measurement pulses is 55 kHz, corresponding to a center position shift of ~0.1 μm. The width of each measurement pulse is set to 0.4 μs, and the free evolution time between consecutive measurements is 1.4 μs. During this free evolution time, the FPGA gradually adjusts the driving signal frequency of the measurement pulses without altering the intensity, ensuring that the measurement pulse intensity remains at zero and does not interfere with the atom’s free evolution.

Figure 5 c shows the experimental result of single-shot atomic fluorescence imaging signals captured by the EMCCD. The pixel size of the EMCCD is 16 μm, and considering the magnification of the imaging system, the actual distance in the plane where the atom stays corresponds to ~1 μm.

## Supplementary information

Transparent Peer Review file

## Data Availability

The data and code supporting the findings of this study have been deposited in a public repository and are openly accessible via Zenodo^57^.
