# Supplementary material for: Quantum Zeno effect in the spatial evolution of a single atom
Source: Nat Commun. 2026 Jun 12;17:7464. doi: 10.1038/s41467-026-74332-1 (PMC13408358; doi:10.1038/s41467-026-74332-1)
Supplement: Supplementary file 1 — Transparent Peer Review file [file 41467_2026_74332_MOESM1_ESM.pdf]

nature portfolio

Peer Review File

Quantum Zeno Effect in the Spatial Evolution of a Single Atom

Corresponding Author: Professor Dongsheng Ding

This file contains all reviewer reports in order by version, followed by all author rebuttals in order by version.

Version 0:

Reviewer comments:

Reviewer #1

(Remarks to the Author)

The topic of this work is sufficiently important to merit a well-founded experimental and theoretical demonstration. However, despite the claim that repeated projective measurements are performed in this work on the moving atomic wavepacket, the dynamics effected by switching on and off the dipole trap is entirely unitary. It is misleading to treat them as "measurements", the only real measurement occurs only at the end of the process when fluorescence is detected, but this is not the cause for the dynamics which should supposedly give rise to the Zeno effect.
The motion is simply describable by a time-dependent Hamiltonian with kinetic energy and alternating potential parts. Clearly, the resulting "stop & go" motion can be slowed down as the number of pulses, their strength and duration increase, in a perfectly unitary fashion.
This does not indicate the occurrence of the Zeno effect caused by measurements, which should break the wavepacket coherence, unlike the method used.
The ability to switch from the Zeno to the anti-Zeno regime as the spacing between pulses (not the single-pulse duration) is varied would be a more convincing proof, but even such a demonstration of motional speed change should be effected by genuine measurements to justify the names Zeno and anti-Zeno.
The quantitative analysis that supposedly interprets the results as projective measurements is based on the approximation that the potential is a quadratic (x^2) function, but this approximation fails far from the trap center. One therefore cannot claim that "the atom is inside" when this approximation holds or "the atom is outside" when it does not. Nothing is reliably measured in this way.
For these reasons the paper cannot be published. The entire approach has to be changed to justify the claims. Unfortunately, I must recommend rejection.

Reviewer #2

(Remarks to the Author)
This manuscript reports a compelling experimental investigation into the Quantum Zeno Effect (QZE) arising from the spatial evolution of a single cold atom in free space. By employing a modulated optical tweezer as a precise projection mechanism, the authors demonstrate the suppression of the atom's free-evolution spreading through frequent position measurements. The work further explores how measurement frequency and strength shape the atomic motion dynamics, including a demonstration of measurement-induced directional transport.

The study represents a substantial contribution to the field of quantum measurement and single-atom control. It offers an intuitive physical picture of measurement backaction in the spatial degree of freedom and suggests a potentially effective route for motional state engineering. The manuscript is well-motivated, clearly described, and the experimental results are highly convincing. I recommend publication in Nature Communications, pending the authors thoroughly address the concerns and questions below.

Q1: The theoretical models provided in the main text and Method section effectively frame the physical picture of the measurement-induced suppression. However, the manuscript currently relies solely on empirical fit curves in the figures, lacking a strong quantitative link between the presented models and the experimental data. So, if possible, could the authors

provide simulation results (theoretical curves) based on your models to accompany the experimental observations in the main figures? A discussion contrasting these models with the experimental data, potentially leveraging semi-classical approaches (e.g., Monte-Carlo simulation of the release-recapture process, referencing works like Phys. Rev. A 78, 033425 (2008)), would significantly strengthen the work.

Q2: The authors should clarify the atom temperature of the single atom before the evolution begins. From the loss probability of ~ 0.8 without measurement pulses in a 30 μs release window in Fig. 3, I assume the temperature should be high and the atom is not cooled to populate the lowest vibrational state of the trap. That means the wavepacket of the single atom should populates a series of high vibrational states of the tweezer trap. I suggest the authors reconsider the correctness of the schematic in Fig. 2b. Also, it should be helpful to discuss the effect of the initial temperature of the single atom, e.g., should such suppression effect still exist for extremely high/low temperatures? From the viewpoint of the thermalization, can such recapture effect still be understood as a kind of quantum Zeno effect?

Q3: Given the finite initial temperature (Question 2), all measured data points represent average values across multiple repeated experimental cycles. The authors should clearly state the typical number of repetition times used to obtain each data point and provide a detailed explanation of the statistical methodology employed.

Q4: In Fig. 3, the baseline loss probability without measurement pulses (zero strength I) differs between panels (a), (b), and (c). Please interpret the variation in this zero-strength loss probability across the panels. Does this indicate the atoms were prepared in different initial conditions or temperatures for each experiment? Also, the authors should clarify to what value the measurement pulse strength I is normalized

Q5: The loss probability curves in Fig. 3 (a), (b), and (c) reach distinct saturation values (I would call them asymptotic convergence thresholds) when the pulse intensity is increased. The authors should provide a detailed interpretation of this saturation phenomenon in the main text. Additionally, is this asymptotic threshold affected by the heating rate of the 840nm trap? The authors should provide information of the scattering/heating rate of the 840nm laser. I guess it should introduce relatively neglectable loss probability compared to the recapture effect.

Q6: The authors should provide information about the trap frequencies. As the trap modulation is performed within several tens of microseconds, should it cause the parametric heating to the single atom? Specifically, is the frequency spectrum resulting from the Fourier transform of the modulation pulses far from or close to the initial trap frequencies?

Q7: Related to question 6, the modulation of the tweezer trap is very similar to the time-dependent 'TOP' trap in cold atom experiments. That means we can understand that, the single atom feels an effective optical trap in the release window. Can the loss probability be directly related to the effective trap depth/frequencies? Is it possible to unify the data dependencies (on modulation frequency/pulse intensity) using the scale of the effective trap depth/frequency? The survival probability should be related to the 'Franck-Condon' overlap of the initial wave packet with the effective trap's vibrational levels. Can the authors provide brief insights into the feasibility of modeling this process using a fully quantum mechanical language?

Q8: Figure 4 shows that the loss is suppressed for short and long pulse durations, but the effect is reduced for the intermediate $3.5 \mu s$ duration. Does this behavior indicate a crossover from the Quantum Zeno to the Anti-Zeno regime? Also, please investigate if the behavior observed at $3.5 \mu s$ relates to the parametric heating discussed in question 6.

Q9: In Fig. 5, the authors show how to control the atomic motion by modulating the trap parameters. Are there any advantages for such pulsed modulation compared to the case by just directly ramping the driving frequency of the AOD adiabatically? The authors claim this method avoids additional momentum transfer compared to adiabatically ramping the AOD driving frequency. So, to support this claim, the authors should measure and show the temperature of the single atom after the directional moving in Fig. 5, comparing it to the initial value. Also, a valuable addition for future applications would be a detailed discussion of the possible limitations of this transport scheme, e.g., maximum moving speed, coherence lifetime, and heating effects.

Q10: Minor comments:
(1) In the introductory discussion of QZE experimental observations (first page), please reference key experiments from other cold atom platforms, such as those involving ultracold molecules [Phys. Rev. Lett. 112, 070404 (2014)], momentum space lattices [npj Quantum Information 7, 78 (2021)], quantum gases [Nat. Commun. 10, 855 (2019), Science 349, 1317 (2015)], and Rydberg atoms [Nat. Phys. 10, 715 (2014)].
(2) I suggest adding a dedicated subsection in the Method section providing more quantitative details about the data analysis, systematic uncertainties, and calibration procedures.
(3) Phrasing check: The phrasing, such as "enabling deterministic preparation of distinct motional states," should be carefully clarified. The word "deterministic" seems ambiguous given the current evidence and may be an overstatement. A careful proofread for typos and minor language issues throughout the manuscript is recommended.

Version 1:

Reviewer comments:

Reviewer #1

(Remarks to the Author)
Review NCOMMS-25-79245A:

The revised version has satisfactorily addressed the outstanding technical issues. However, my main objection has remained unanswered. The protocol describes a sequence of unitary operations caused by alternating Hamiltonians but not projective measurements. Thus the connection to the QZE is inappropriate.

A secondary remark is that if this were the QZE, then there should be also the AZE for other parameters. However, this does not seem to be the case. The paper cannot be published on the basis of the material and interpretation at hand. I can only reiterate that the paper is conceptually flawed and does not describe the quantum Zeno effect. The same objections remain as in my first report and the new version does not address them.

Reviewer #2

(Remarks to the Author)
The authors address all related questions properly, and now I recommend publication.

I have a different view regarding the point raised by Reviewer #1 that "the dynamics effected by switching on and off the dipole trap is entirely unitary. It is misleading to treat them as measurements." In my opinion, the results presented clearly demonstrate that the process of turning on the dipole trap constitutes a measurement of the spatial position of the atomic motional wavefunction. Sometimes a phenomenon can be interpreted/treated with different physical pictures, no matter the measurements/projections or the loss to the reservoir can all lead to the quantum Zeno effect. I think this work directly relates to the quantum Zeno dynamics described in an old paper [Phys. Rev. Lett. 89, 080401 (2002)].

Specifically, when the duration of the dipole trap activation exactly matches the oscillation period of the atom within the trap (corresponding to the data in Fig. 2(c)), a high frequency of trap activation leads to a reduction in the atomic loss probability. If the process could be fully described by unitary evolution under a time-dependent Hamiltonian, the final motional state of the atomic wavefunction and the observed loss probability should remain unchanged regardless of how many complete oscillation cycles occur under the influence of the dipole trap, provided the total free evolution time is the same. This directly contradicts the phenomenon observed in this work that high-frequency activation of the dipole trap suppresses atomic motion in real space and instead aligns with the process described by the quantum Zeno dynamics, demonstrating that the trap activation functions as a positional projection measurement.

Open Access This Peer Review File is licensed under a Creative Commons Attribution 4.0 International License, which permits use, sharing, adaptation, distribution and reproduction in any medium or format, as long as you give appropriate credit to the original author(s) and the source, provide a link to the Creative Commons license, and indicate if changes were made.
In cases where reviewers are anonymous, credit should be given to 'Anonymous Referee' and the source.
The images or other third party material in this Peer Review File are included in the article’s Creative Commons license, unless indicated otherwise in a credit line to the material. If material is not included in the article’s Creative Commons

license and your intended use is not permitted by statutory regulation or exceeds the permitted use, you will need to obtain permission directly from the copyright holder.
To view a copy of this license, visit https://creativecommons.org/licenses/by/4.0/

REVIEWER COMMENTS

Reviewer #1:

The topic of this work is sufficiently important to merit a well-founded experimental and theoretical demonstration.

Reply:

We sincerely thank the referee for the positive assessment of the importance of the topic studied in this work and for acknowledging that it merits careful experimental and theoretical investigation.

Major Questions:

1. However, despite the claim that repeated projective measurements are performed in this work on the moving atomic wavepacket, the dynamics effected by switching on and off the dipole trap is entirely unitary. It is misleading to treat them as "measurements", the only real measurement occurs only at the end of the process when fluorescence is detected, but this is not the cause for the dynamics which should supposedly give rise to the Zeno effect.

Reply:

We thank the referee for the careful reading of our manuscript and for raising a crucial question regarding the physical meaning of "measurement" in our work. We would like to provide the following clarification.

In our experiment, the periodic switching on of the optical tweezer trap does not introduce a purely unitary dynamical process alone. Rather, it intrinsically involves two inseparable components: a measurement process and the trap-induced unitary evolution. Specifically, when the trap is switched on, the full set of bound eigenstates $\{|b_n\rangle\}$ of the trap Hamiltonian defines an effective projection subspace. This operation implements a conditional projection on whether the atomic wavefunction occupies the position–momentum region that can be supported by the trap, i.e., it

constitutes a measurement of whether the atom is "inside the trap." In this process, the component of the wavefunction projected onto the bound-state subspace continues to participate in the subsequent dynamics, whereas the component orthogonal to this subspace couples to the continuum of free states, corresponding to atom loss observed in the experiment. We will discuss this complete physical process in more detail in the following section (Q2), where we also show, based on key experimental observations—that a description relying solely on fully unitary dynamics is insufficient to account for the observed behavior induced by the trap switching.

At the same time, as correctly pointed out by the referee, the fluorescence imaging performed at the end of the experimental sequence also constitutes a measurement, corresponding to a position measurement of "where the atom is." Although both processes involve position-related measurements, they play fundamentally different roles in the experiment. The trap-associated measurement directly participates in and shapes the atomic motion and survival dynamics throughout the sequence, whereas the fluorescence imaging occurs only after the dynamical evolution has concluded, serving to read out and record the atomic position via its interaction with photons. Therefore, regarding the final fluorescence detection as the only "real measurement" in the experiment does not fully capture the measurement backaction structure inherently present in the experimental protocol.

Manuscript change:

- We have added a more detailed description of the physical processes in the manuscript：

  "The atom is first released from the optical tweezer and enters the vacuum chamber, where it undergoes a period of unitary evolution in free space governed by the free-particle Hamiltonian $H_{\text{free}} = P^2/(2m)$. Subsequently, a Gaussian-shaped trapping potential is switched on again and maintained for a finite duration τ . Physically, the switching-on of the trap contains two inseparable ingredients. The first is a measurement process that determines

whether the atomic wavefunction still lies within the bound-state subspace of the trapping potential. The second is the subsequent unitary evolution under the trap Hamiltonian:

$$H_{\text{trap}} = H_{\text{free}} - U_0 \exp(\frac{-2r^2}{\omega_0{}^2}),$$

where U_0 denotes the trap depth, r is the distance of the atom from the trap center, and ω_0 is the beam waist of the optical tweezer. If the atom is not lost during this measurement process, its wavefunction continues to participate in the subsequent dynamics. After the trap is switched off again, the atom is released and enters the next experimental cycle. After completing the full sequence of measurements, the atomic position is detected via fluorescence imaging at the trap location, which determines whether the atom ultimately remains trapped."

2. The motion is simply describable by a time-dependent Hamiltonian with kinetic energy and alternating potential parts. Clearly, the resulting "stop & go" motion can be slowed down as the number of pulses, their strength and duration increase, in a perfectly unitary fashion. This does not indicate the occurrence of the Zeno effect caused by measurements, which should break the wavepacket coherence, unlike the method used.

Reply:

We thank the referee for the careful and in-depth analysis of this issue. We understand that the referee points out that, at a formal level, the motion of an atom subject to a periodically switched-on and switched-off trap may appear to be describable by a time-dependent Hamiltonian consisting of a kinetic-energy term and a time-modulated potential term, leading to a purely unitary "stop-and-go" dynamics. Within such a framework, it is indeed natural that the atomic motion becomes increasingly slowed as the number, strength, and duration of the pulses are increased. However, we must emphasize that this purely unitary description neither adequately

nor correctly captures the essential dynamics of the present experiment, and it is fundamentally inconsistent with several key experimental observations.

From a physical point of view, the experimental sequence consists of the following elements. The atom is first released from the optical tweezer and undergoes a period of unitary evolution governed by the free-space Hamiltonian $H_{\text{free}} = P^2/(2m)$. Subsequently, a Gaussian trapping potential is switched on for a finite duration τ. This process contains two inseparable ingredients: a measurement that determines whether the atomic wavefunction still lies within the bound-state subspace of the trap, and a unitary evolution under the trap Hamiltonian $H_{\text{trap}} = H_{\text{free}} - U_0 \exp(\frac{-2r^2}{\omega_0{}^2})$, where U_0 is the trap depth, r the distance from the trap center, and ω_0 the beam waist. During this non-unitary measurement process, the atomic wavefunction is effectively tested against the bound-state subspace of the trap. If the atom is recaptured, its wavefunction is conditionally restricted to this bound-state subspace and continues to participate in subsequent dynamics. If it is not recaptured, the wavefunction irreversibly couples to the continuum of free states, corresponding to atomic loss. This loss channel constitutes the physical basis for the emergence of the quantum Zeno effect in our system. If no loss occurs during a given measurement, the atom proceeds to the subsequent stages of the experimental sequence. Therefore, the periodic switching of the optical tweezer is not equivalent to merely introducing a time-modulated potential term into the Hamiltonian. Treating the entire process as a continuous unitary evolution generated by a time-dependent Hamiltonian represents an oversimplification of the actual experimental dynamics.

Based on this physical picture, we constructed a theoretical model consisting of a repeated sequence of release, free evolution, measurement, and in-trap evolution, and performed Monte Carlo simulations to quantitatively describe the measurement-induced suppression of spatial evolution. In the experiment, a single atom is initially released from a Gaussian trap with depth $U_0 = 2\,m\text{K}$ and beam

waist $\omega_0 = 1.2\ \mu\text{m}$; the same parameters are used in the simulations. To account for the finite atomic temperature ($T_{\text{atom}} = 175\ \mu\text{K}$, measured following the method of Phys. Rev. A 78, 033425 (2008)), the initial wavefunction $|\phi_0\rangle$ is randomly sampled from all bound states according to a Boltzmann distribution,

$$P_n \propto \exp(-E_n / k_B T_{\text{atom}})$$

where E_n is the eigenenergy of the n -th bound state of H_{trap} and k_B is the Boltzmann constant. After the trap is turned off, the atom evolves freely for a time t, resulting in a wavefunction $|\phi_t\rangle$. When the trap is switched on again, a projection measurement onto the bound-state subspace $\{|b_n\rangle\}$ is performed: with probability $p_n = |\langle b_n|\phi_t\rangle|^2$, the wavefunction is updated to the corresponding bound state $|b_n\rangle$; if the wavefunction does not project onto any bound state, the atom is defined as lost in that measurement, yielding a loss probability $P_{\text{loss}} = 1 - \sum_n |\langle b_n|\phi_t\rangle|^2$. In the numerical simulations, the measurement back-action is implemented via a stochastic process, where random numbers determine the wavefunction update. If the atom survives the measurement, the updated wavefunction evolves under H_{trap} for a duration τ , after which the next cycle begins.

After the full measurement sequence is completed, the experiment detects the atomic position via fluorescence imaging to determine whether the atom remains trapped. Correspondingly, in the simulations, the outcome of the final stochastic process determines whether the atom remains within the bound-state subspace. Repeating this procedure sufficiently many times N , we obtain the number of surviving trajectories N_{surv} , from which the final loss probability $1\text{-N}_{\text{surv}} / \text{N}$ is extracted. Experimentally, each parameter set is repeated 500 times and averaged over three independent datasets. Due to the probabilistic loading of single atoms from the magneto-optical trap (MOT), the effective number of realizations per dataset is

approximately 300. In the Monte Carlo simulations, we directly use 500 trajectories. We emphasize that the stochastic process employed in the Monte Carlo simulations is not a numerical artifact, but a direct representation of the physically real measurement process in the experiment. If this stochastic measurement process is artificially removed and only the unitary evolution induced by the time-modulated trap is retained, the results exhibit systematic deviations from the experimental observations, as shown below.

A direct and crucial piece of evidence that trap switching includes a measurement process is provided by the non-zero saturation loss probability observed in Fig. 3. Experimentally, as the trap depth used in the measurement pulses is increased, the atomic loss probability does not monotonically approach zero, but instead enters a saturation regime beyond a certain threshold, with the saturation value decreasing as the measurement frequency increases. In our model, this behavior has a clear physical interpretation: when the trap depth becomes sufficiently large, a short trap pulse already approximates an ideal projective measurement, and further increasing the measurement strength does not significantly alter the single-shot projection outcome. More specifically, increasing the trap depth enlarges the effective dimension of the bound-state subspace and increases its overlap with the post-evolution wavefunction. However, due to the relative orthogonality between high-dimensional bound-state subspaces and the atomic wavefunction at a given temperature, this overlap cannot increase indefinitely to unity. Consequently, the probability of recapture in a single measurement has an upper bound, leading to a saturation of the cumulative loss probability after many measurements.

Such behavior is impossible within any purely unitary “stop-and-go” model. In a description based solely on a time-dependent Hamiltonian, increasing the trap strength can only monotonically enhance the effective confinement, causing the loss probability to decrease continuously and asymptotically approach zero, rather than saturating at a finite value. This contrast is explicitly demonstrated in Supplementary Fig. SA1, where all parameters are identical to those used in Fig. 3(a) of the main text. In the purely unitary model (orange curve), obtained by removing the measurement

process, the loss probability decreases monotonically to zero with increasing trap depth. In contrast, the model including measurements (blue curve) exhibits a clear non-zero saturation around 0.4, in agreement with the experimental observations. Minor non-monotonic fluctuations arise from Monte Carlo statistical noise. Therefore, the non-zero saturation observed in Fig. 3 itself constitutes an experimental fact that cannot be explained by purely unitary dynamics, but can only be understood by explicitly incorporating measurement-induced non-unitarity.

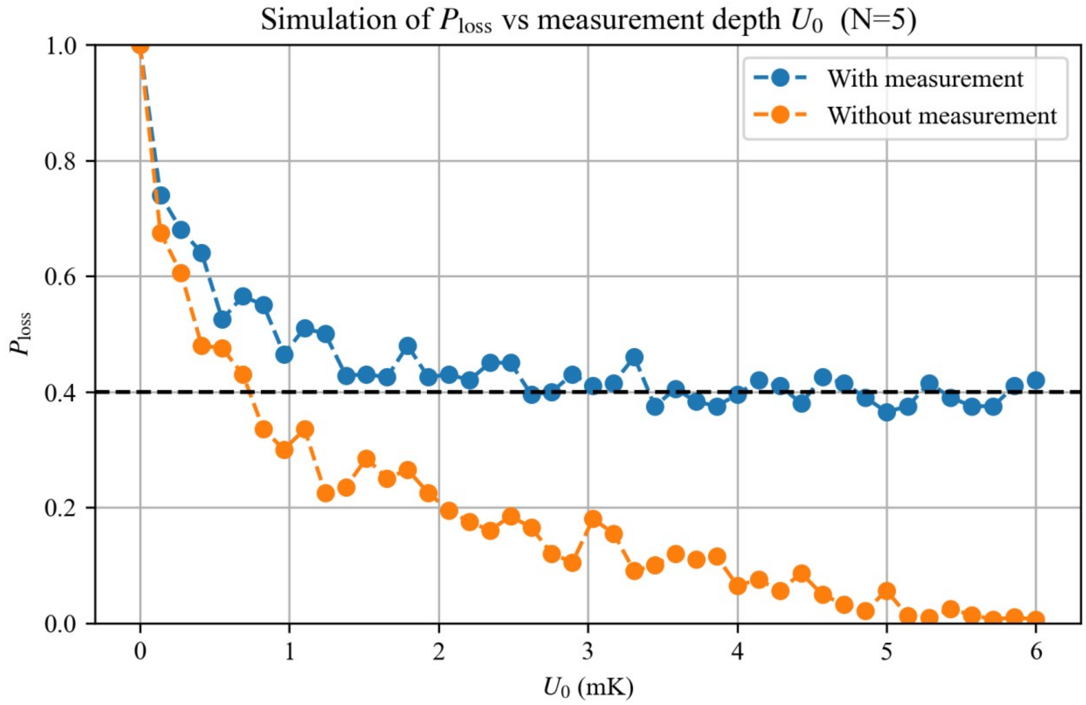

Supplementary Fig. SA1

Further decisive evidence is provided by the results shown in Fig. 5. As discussed in the manuscript, the effective atomic drift velocity achieved in our experiment is approximately $0.7\, m/s$, which is significantly higher than the maximum stable velocity (about $0.05\, m/s$) achievable via trap dragging in the same system under comparable parameters (continuous trap, gradually chirped AOD frequency). Such a velocity is likewise incompatible with a purely unitary description. In any unitary transport process based on trap dragging, the atomic velocity is necessarily limited by adiabaticity; exceeding this limit leads to severe loss due to the breakdown of confinement. Consequently, within a purely unitary framework, the effective transport

velocity cannot exceed the maximum dragging velocity allowed by a continuously on trap. This clearly demonstrates that the spatial displacement in our experiment does not arise from continuous trap dragging, but rather from measurement back-action itself: each measurement re-selects and effectively "resets" the atomic spatial distribution at a new position. This mechanism is fundamentally distinct from any unitary dragging process and removes the adiabaticity constraint on the effective transport speed.

In summary, although a time-dependent Hamiltonian can formally be written to describe the trap switching, reducing the experimental dynamics to a purely unitary motion is physically insufficient. Both the non-zero saturation behavior observed in Fig. 3 and the super-adiabatic effective transport velocity observed in Fig. 5 unambiguously demonstrate that only by explicitly incorporating measurement-induced non-unitary dynamics can the experimental results be consistently and reasonably explained.

Manuscript change:

- We have refined the theoretical part of the manuscript and incorporated the calculation results into the revised figures:

  "Based on the physical picture described above, we construct a theoretical model in which the atomic dynamics are described by a repeated four-stage sequence: release from the trap, free evolution, measurement, and subsequent in-trap evolution. Using exactly the same parameters as in the experiment, we then perform quantitative Monte Carlo simulations of the atomic dynamics. In the experiment, a single atom is initially released from a Gaussian optical trap with depth $U_0 = 2\,m\mathrm{K}$ and beam waist $\omega_0 = 1.2\,\mu\mathrm{m}$. Taking into account the finite atomic temperature $T_{\mathrm{atom}} = 175\,\mu\mathrm{K}$ in the trap, the initial state $|\phi_0\rangle$ is described as a thermal mixed state obeying the corresponding statistical distribution. In the simulations, the $|\phi_0\rangle$ is generated by randomly sampling all bound states of the trapping potential according to the Boltzmann distribution.

Specifically, the probability for the atom to occupy the n-th bound state $|b_n\rangle$ of the trapping Hamiltonian H_{trap} is given by:

$$P_n \propto \exp(-E_n / k_B T_{\text{atom}}),$$

where E_n is the eigenenergy of $|b_n\rangle$, and k_B is the Boltzmann constant."

"After release, the atom undergoes free-space evolution U for a duration t, after which the optical tweezer trap is switched on again. At this moment, the atomic wavefunction $|\phi_t\rangle$ is subjected to a projective measurement in the basis of all bound eigenstates $\{|b_i\rangle\}$ of the trap. The atom collapses into a specific bound state with probability $p_n = |\langle b_n|\phi_t\rangle|^2$, while the total probability that the atom is not captured by any bound state is defined as the loss probability during this measurement, denoted by $P_{\text{loss}} = 1 - \sum_n |\langle b_n|\phi_t\rangle|^2$. In the numerical simulations, the measurement back-action is implemented through a stochastic sampling procedure, where the outcome of each measurement is determined by random number generation. If the atom survives the measurement, the updated wavefunction subsequently evolves under the trap Hamiltonian for a duration τ, after which the system enters the next cycle."

"After completing the full sequence of measurements, the experiment determines whether the atom remains trapped via fluorescence imaging. Correspondingly, in the numerical simulations, the outcome of each stochastic process determines whether the atom ultimately remains in the trap. By repeating the above procedure for a sufficiently large number (N_{surv}) of independent realizations, we count the number of surviving events N_{surv}, from which the atomic loss probability $\text{P}_{\text{loss}}=1\text{-N}_{\text{surv}} / \text{N}_{\text{total}}$ is extracted. Experimentally, each data set consists of 500 repeated experimental runs, and each data point is obtained by averaging over three independent data sets under identical parameters. Due to the probabilistic loading of single atoms from the MOT, the effective number of

realizations per data set is approximately 300. In the Monte Carlo simulations, we directly use 500 trajectories."

"In the short-pulse regime, the action of a measurement pulse on the atomic motional state, denoted as $M(\tau_1)$, can be well approximated by an ideal projective measurement onto the bound-state subspace, $M_{\mathrm{b}} = \sum_n |\mathrm{b}_n\rangle\langle \mathrm{b}_n|$. Under this action, the atomic wavefunction becomes primarily localized within the spatial region covered by the Gaussian trapping potential after the pulse, with only minor leakage along the direction of the initial group velocity. Once the trap is switched off, the wavefunction continues to expand into free space, as illustrated in Fig. 1(b1). Considering that, during a single free-evolution interval of duration T/N, the atomic loss probability is given by:

$$\sum_n |\langle \mathrm{b}_n | H_{\mathrm{free}} \, |\psi_0\rangle|^2 \times \mathrm{T}^2 / (\hbar^2 \mathrm{N}^2),$$

the cumulative loss probability after N measurements is therefore proportional to $1/\mathrm{N}$ (see Methods for details). The corresponding experimental results are shown by the red data points with error bars in Fig. 1(c): as the number of measurements increases, the loss probability rapidly approaches zero following an inverse dependence, indicating that frequent projective measurements effectively freeze the spatial evolution of a single atom, giving rise to the QZE. Monte Carlo simulations of the same process, shown as lighter red dots, exhibit good agreement with the experimental trend."

"In contrast, in the long-pulse regime, the wavefunction undergoes substantial unitary evolution inside the trap following the collapse induced by the measurement (Fig. 1(b2)). In this case, the action of a measurement pulse on the atomic motional state can be expressed as:

$$M(\tau_2) = e^{-iH_{\mathrm{trap}}\tau_2/\hbar} M_{\mathrm{b}},$$

which leads to a larger fraction of the wavefunction components extending beyond the trapping region at the end of a single pulse. This behavior arises because, during the evolution inside the trap, the atomic wavefunction acquires an

additional phase distribution, causing its subsequent free-space evolution to depend sensitively on the residence time within the trap. This effect is reflected in the blue curve in Fig. 1(c). For $\tau = 5.0?\mu s$, the duration of the measurement pulse corresponds to one half of the trap oscillation period. In an idealized picture, the unitary evolution of the atomic wavefunction inside the trap drives its spatial distribution back toward a localized state near the trap center at the end of the pulse, such that the atomic loss probability remains at a relatively low level (see the next section). However, the above discussion primarily addresses atom loss arising from the reduced overlap between the atomic wavefunction and the bound-state subspace. In the actual experiment, coupling between the atom and the environment introduces additional loss channels. The increased total evolution time associated with long pulses therefore leads to a noticeably higher loss probability at low measurement frequencies compared with the short-pulse case. As the measurement frequency is increased, the measurement-induced quantum Zeno effect gradually becomes dominant, and the atomic loss probability correspondingly decreases with increasing measurement frequency."

3. The ability to switch from the Zeno to the anti-Zeno regime as the spacing between pulses (not the single-pulse duration) is varied would be a more convincing proof, but even such a demonstration of motional speed change should be effected by genuine measurements to justify the names Zeno and anti-Zeno.

Reply:

We thank the reviewer for the constructive suggestion of demonstrating a crossover from the Quantum Zeno to the anti-Zeno regime by varying the spacing between measurement pulses (rather than the duration of individual pulses). We agree that, in systems where the relevant physical conditions are satisfied, observing a Zeno-anti-Zeno crossover can indeed provide compelling evidence to distinguish between the two effects. However, we emphasize that within the parameter regime explored in the present experiment, the anti-Quantum Zeno effect is not physically

accessible. Consequently, the absence of an observed anti-Zeno regime cannot be taken as evidence against the presence of genuine quantum measurements in the experimental process.

The anti-Quantum Zeno effect typically arises when the characteristic frequency scale introduced by the measurement process significantly overlaps with the spectral density of the system's decay channels into continuum states (or the environment), thereby enhancing the effective decay rate. In our experimental system, atom loss from the optical tweezer predominantly corresponds to coupling into unbound free-space continuum states. The relevant energy scale of this process is determined by the difference between the trap depth and the atomic kinetic energy. For the trap parameters used in this work (with a trap depth of approximately $2\ \mathrm{mK}$), this energy scale corresponds to a characteristic frequency on the order of tens of MHz. In contrast, the frequency scale associated with the measurement cycle in our experiment lies only in the range of several tens to a few hundred kHz. Even at the shortest pulse spacing permitted by the experimental setup, this frequency remains several orders of magnitude smaller than the dominant spectral weight associated with coupling to the continuum states. Therefore, within this parameter regime, varying the spacing between measurement pulses can only modulate the strength of the measurement-induced suppression, but cannot induce a transition from suppressed to enhanced dynamics. Experimentally, we consistently observe a characteristic Quantum Zeno–type dynamical response, and adjusting the measurement pulse spacing alone is insufficient to drive the system into the anti-Quantum Zeno regime.

In summary, although in principle a Zeno-anti-Zeno crossover may be achieved by tuning the measurement interval in suitable systems, such a crossover is physically inaccessible within the frequency and energy scales explored in the present experiment. The absence of an observed anti-Quantum Zeno effect therefore does not undermine our identification of the observed dynamics as measurement-induced Quantum Zeno behavior.

Manuscript change:

- The manuscript has not been modified in this respect.

3. The quantitative analysis that supposedly interprets the results as projective measurements is based on the approximation that the potential is a quadratic (x^2) function, but this approximation fails far from the trap center. One therefore cannot claim that "the atom is inside" when this approximation holds or "the atom is outside" when it does not. Nothing is reliably measured in this way.

Reply:

We thank the reviewer for raising this important point concerning the validity range of the quadratic (harmonic) approximation of the trapping potential. We agree that a Gaussian optical tweezer can be approximated by a quadratic (harmonic oscillator) potential only in the vicinity of the trap center, and that this approximation breaks down at larger distances. At the same time, we note the reviewer’s statement that “one therefore cannot claim that ‘the atom is inside’ when this approximation holds or ‘the atom is outside’ when it does not.” We would like to emphasize that no such statement or logical assumption is made anywhere in the manuscript. In the original version, we never associate the criterion of “atom being inside/outside the trap” with the validity of the harmonic approximation, nor do we base the measurement of any physical quantity on whether this approximation holds.

In our experiment, the physical quantity being measured is not whether the atom resides in a region where the trapping potential can be approximated as harmonic. Instead, it is a fundamentally different and more basic criterion: whether the atom remains bound to the optical tweezer or has irreversibly escaped from it. As discussed in the manuscript, this “recapture versus loss” outcome corresponds to a real physical process in the experiment and is determined by the overlap between the atomic wave function and the bound-state subspace of the trap. This process does not rely on whether the potential can be locally approximated by a quadratic form.

In this sense, the measurement implemented in the experiment is a genuine, physically observable measurement of whether the atom remains trapped, rather than

a formal classification based on the validity of a local potential approximation. The associated non-unitary dynamics arise from the irreversible measurement backaction itself, and their physical origin is independent of whether the trapping potential admits a harmonic approximation.

Furthermore, to eliminate any possible ambiguity introduced by approximate potential models, we have replaced all calculations based on the harmonic approximation with numerical simulations using the full Gaussian optical tweezer potential, including an explicit treatment of the bound-state and continuum structure as well as the atomic loss process. We find that the resulting dynamical behavior, the measurement-induced suppression effect, and the agreement with experimental data remain unchanged. This demonstrates that our conclusions regarding measurement-induced quantum Zeno dynamics do not rely on the use of the harmonic approximation.

Manuscript change:

- We have clarified the corresponding descriptions in the revised manuscript to ensure that the physical meaning is conveyed more clearly and to avoid any confusion between model approximations and experimentally observable criteria.

4. For these reasons the paper cannot be published. The entire approach has to be changed to justify the claims. Unfortunately, I must recommend rejection.

Reply:

We thank the referee for the careful examination of the physical issues addressed in the manuscript and fully respect the referee's views regarding the overall approach and physical interpretation of this work. In response to the referee's main concerns, we have provided detailed clarifications in the preceding replies regarding the physical picture underlying the experimental procedure. We sincerely hope that these additional explanations help to further clarify the motivation and results of our work, and we respectfully ask the referee to reconsider the overall evaluation of the manuscript in light of these clarifications.

==

REVIEWER COMMENTS

Reviewer #2:

This manuscript reports a compelling experimental investigation into the Quantum Zeno Effect (QZE) arising from the spatial evolution of a single cold atom in free space. By employing a modulated optical tweezer as a precise projection mechanism, the authors demonstrate the suppression of the atom's free-evolution spreading through frequent position measurements. The work further explores how measurement frequency and strength shape the atomic motion dynamics, including a demonstration of measurement-induced directional transport.

Reply:

We thank the referee for the careful evaluation of our manuscript and for the constructive comments on the clarity, significance, and experimental details of our work. We have carefully considered all comments and revised the manuscript accordingly.

Major Questions:

1. The theoretical models provided in the main text and Method section effectively frame the physical picture of the measurement-induced suppression. However, the manuscript currently relies solely on empirical fit curves in the figures, lacking a strong quantitative link between the presented models and the experimental data. So, if possible, could the authors provide simulation results (theoretical curves) based on your models to accompany the experimental observations in the main figures? A discussion contrasting these models with the experimental data, potentially leveraging semi-classical approaches (e.g., Monte-Carlo simulation of the release-recapture process, referencing works like Phys. Rev. A 78, 033425 (2008)), would significantly strengthen the work.

Reply:

We thank the referee for this constructive suggestion. Following the referee's recommendation, we have further extended our theoretical framework by incorporating Monte Carlo simulations, thereby significantly strengthening the quantitative connection between the theoretical models and the experimental data.

Within the refined physical framework, the dynamics of a single atom undergoing repeated position measurements is modeled as a release–measurement-sequence–recapture process. The Monte Carlo simulations are based on the same physical assumptions as the theoretical models introduced in the main text and Methods, and are used to quantitatively describe the measurement-induced suppression of spatial evolution.

Specifically, the atom is initially released from the trapping potential, and its initial state $|\phi_0\rangle$ is chosen as one of the bound eigenstates of a Gaussian optical trap. To account for the finite temperature of the atom in the trap, the initial bound state is randomly sampled from all bound eigenstates according to a Boltzmann distribution

$$P_n \propto \exp(-E_n / k_B T_{\text{atom}}),$$

where $E_n < 0$ denotes the eigenenergy of the corresponding bound state, T_{atom} is the effective atomic temperature in the trap, and k_B is the Boltzmann constant.

After release, the atom evolves freely in space for a duration t, after which the trapping potential is switched on again. At this moment, the atomic wavefunction $|\phi_t\rangle$ undergoes a projective measurement in the basis of all bound eigenstates $\{|b_n\rangle\}$. The atom collapses into a given bound state with probability $p_n = |\langle b_n | \phi_t \rangle|^2$, while the total probability that the atom is not captured by any bound state is defined as the loss probability during this measurement $P_{\text{loss}} = 1 - \sum_n |\langle b_n | \phi_t \rangle|^2$.

In the numerical simulations, this measurement process is implemented through a stochastic sampling procedure. To reduce the computational complexity while retaining the essential physical behavior, we effectively project the atom back to its

initial bound state whenever it is not lost, which captures the repeated confinement of the atom near the trap center under frequent measurements.

If the atom remains trapped after the measurement, its wavefunction subsequently evolves under the trapping Hamiltonian H_{trap} for a duration τ, followed by the next release–free evolution–measurement cycle. After completing the full sequence of measurements, the atomic wavefunction evolves to a final state $|\phi_{\mathrm{f}}\rangle$. In the experiment, the survival of the atom in the trap is detected via fluorescence imaging; correspondingly, in the simulations, a stochastic process is used to determine whether the atom remains in the bound-state subspace.

By repeating the above dynamical process for a sufficiently large number of trajectories (N), we count the number of events $\mathrm{N}_{\mathrm{surv}}$ in which the atom remains trapped at the end of the sequence, yielding a total loss probability: $1\text{-}\mathrm{N}_{\mathrm{surv}}/\mathrm{N}$ .The effective atomic temperature in the trap is estimated using a semi-classical approach, following the method described in Phys. Rev. A 78, 033425 (2008).

The resulting Monte Carlo simulation results are now included as theoretical reference curves in the main figures and are directly compared with the experimental data. Although some quantitative differences remain, the simulations reproduce the overall trends and the parameter dependence observed experimentally across the accessible parameter regime.

Manuscript change:

- Corresponding modifications have been made to the theoretical sections of the manuscript, and the simulation results have been added to the figures for direct comparison with the experimental data:

  "The atom is first released from the optical tweezer and enters the vacuum chamber, where it undergoes a period of unitary evolution in free space governed by the free-particle Hamiltonian $H_{\mathrm{free}} = P^2/(2m)$. Subsequently, a Gaussian-shaped trapping potential is switched on again and maintained for a

finite duration τ . Physically, the switching-on of the trap contains two inseparable ingredients. The first is a measurement process that determines whether the atomic wavefunction still lies within the bound-state subspace of the trapping potential. The second is the subsequent unitary evolution under the trap Hamiltonian:

$$H_{\text{trap}} = H_{\text{free}} - U_0 \exp(\frac{-2r^2}{\omega_0{}^2}),$$

where U_0 denotes the trap depth, r is the distance of the atom from the trap center, and ω_0 is the beam waist of the optical tweezer. If the atom is not lost during this measurement process, its wavefunction continues to participate in the subsequent dynamics. After the trap is switched off again, the atom is released and enters the next experimental cycle. After completing the full sequence of measurements, the atomic position is detected via fluorescence imaging at the trap location, which determines whether the atom ultimately remains trapped."

"Based on the physical picture described above, we construct a theoretical model in which the atomic dynamics are described by a repeated four-stage sequence: release from the trap, free evolution, measurement, and subsequent in-trap evolution. Using exactly the same parameters as in the experiment, we then perform quantitative Monte Carlo simulations of the atomic dynamics. In the experiment, a single atom is initially released from a Gaussian optical trap with depth $U_0 = 2\,m\text{K}$ and beam waist $\omega_0 = 1.2\,\mu\text{m}$. Taking into account the finite atomic temperature $T_{\text{atom}} = 175\,\mu\text{K}$ in the trap, the initial state $|\phi_0\rangle$ is described as a thermal mixed state obeying the corresponding statistical distribution. In the simulations, the $|\phi_0\rangle$ is generated by randomly sampling all bound states of the trapping potential according to the Boltzmann distribution. Specifically, the probability for the atom to occupy the n-th bound state $|b_n\rangle$ of the trapping Hamiltonian H_{trap} is given by:

$$P_n \propto \exp(-E_n / k_B T_{\text{atom}}),$$

where E_n is the eigenenergy of $|b_n\rangle$, and k_B is the Boltzmann constant."

"After release, the atom undergoes free-space evolution U for a duration t, after which the optical tweezer trap is switched on again. At this moment, the atomic wavefunction $|\phi_t\rangle$ is subjected to a projective measurement in the basis of all bound eigenstates $\{|b_i\rangle\}$ of the trap. The atom collapses into a specific bound state with probability $p_n = |\langle b_n|\phi_t\rangle|^2$, while the total probability that the atom is not captured by any bound state is defined as the loss probability during this measurement, denoted by $P_{\text{loss}} = 1 - \sum_n |\langle b_n|\phi_t\rangle|^2$. In the numerical simulations, the measurement back-action is implemented through a stochastic sampling procedure, where the outcome of each measurement is determined by random number generation. If the atom survives the measurement, the updated wavefunction subsequently evolves under the trap Hamiltonian for a duration τ, after which the system enters the next cycle."

"In the short-pulse regime, the action of a measurement pulse on the atomic motional state, denoted as $M(\tau_1)$, can be well approximated by an ideal projective measurement onto the bound-state subspace, $M_{\text{b}} = \sum_n |\text{b}_n\rangle\langle\text{b}_n|$. Under this action, the atomic wavefunction becomes primarily localized within the spatial region covered by the Gaussian trapping potential after the pulse, with only minor leakage along the direction of the initial group velocity. Once the trap is switched off, the wavefunction continues to expand into free space, as illustrated in Fig. 1(b1). Considering that, during a single free-evolution interval of duration T/N, the atomic loss probability is given by:

$$\sum_n |\langle \text{b}_n|H_{\text{free}}|\psi_0\rangle|^2 \times \text{T}^2/(\hbar^2\text{N}^2),$$

the cumulative loss probability after N measurements is therefore proportional to $1/\text{N}$ (see Methods for details). The corresponding experimental results are shown by the red data points with error bars in Fig. 1(c): as the number of

measurements increases, the loss probability rapidly approaches zero following an inverse dependence, indicating that frequent projective measurements effectively freeze the spatial evolution of a single atom, giving rise to the QZE. Monte Carlo simulations of the same process, shown as lighter red dots, exhibit good agreement with the experimental trend."

"In contrast, in the long-pulse regime, the wavefunction undergoes substantial unitary evolution inside the trap following the collapse induced by the measurement (Fig. 1(b2)). In this case, the action of a measurement pulse on the atomic motional state can be expressed as:

$$M(\tau_2) = e^{-iH_{\mathrm{trap}}\tau_2/\hbar} M_{\mathrm{b}},$$

which leads to a larger fraction of the wavefunction components extending beyond the trapping region at the end of a single pulse. This behavior arises because, during the evolution inside the trap, the atomic wavefunction acquires an additional phase distribution, causing its subsequent free-space evolution to depend sensitively on the residence time within the trap. This effect is reflected in the blue curve in Fig. 1(c). For $\tau = 5.0\ \mu\mathrm{s}$, the duration of the measurement pulse corresponds to one half of the trap oscillation period. In an idealized picture, the unitary evolution of the atomic wavefunction inside the trap drives its spatial distribution back toward a localized state near the trap center at the end of the pulse, such that the atomic loss probability remains at a relatively low level (see the next section). However, the above discussion primarily addresses atom loss arising from the reduced overlap between the atomic wavefunction and the bound-state subspace. In the actual experiment, coupling between the atom and the environment introduces additional loss channels. The increased total evolution time associated with long pulses therefore leads to a noticeably higher loss probability at low measurement frequencies compared with the short-pulse case. As the measurement frequency is increased, the measurement-induced quantum Zeno effect gradually becomes dominant, and the atomic loss probability correspondingly decreases with increasing measurement frequency."

2. The authors should clarify the atom temperature of the single atom before the evolution begins. From the loss probability of ~ 0.8 without measurement pulses in a 30 μs release window in Fig. 3, I assume the temperature should be high and the atom is not cooled to populate the lowest vibrational state of the trap. That means the wavepacket of the single atom should populates a series of high vibrational states of the tweezer trap. I suggest the authors reconsider the correctness of the schematic in Fig. 2b. Also, it should be helpful to discuss the effect of the initial temperature of the single atom, e.g., should such suppression effect still exist for extremely high/low temperatures? From the viewpoint of the thermalization, can such recapture effect still be understood as a kind of quantum Zeno effect?

Reply:

We thank the referee for raising the important question regarding the initial temperature of the single atom and its role in the recapture dynamics. In our experiment, the temperature of the single atom before the evolution was first determined using a semi-classical release–recapture thermometry method, as shown in Supplementary Fig. SB1, yielding a value of 175 μK. This temperature indicates that the atom was not cooled to the vibrational ground state of the optical tweezer, but rather occupied a thermal mixture of several trap vibrational levels.

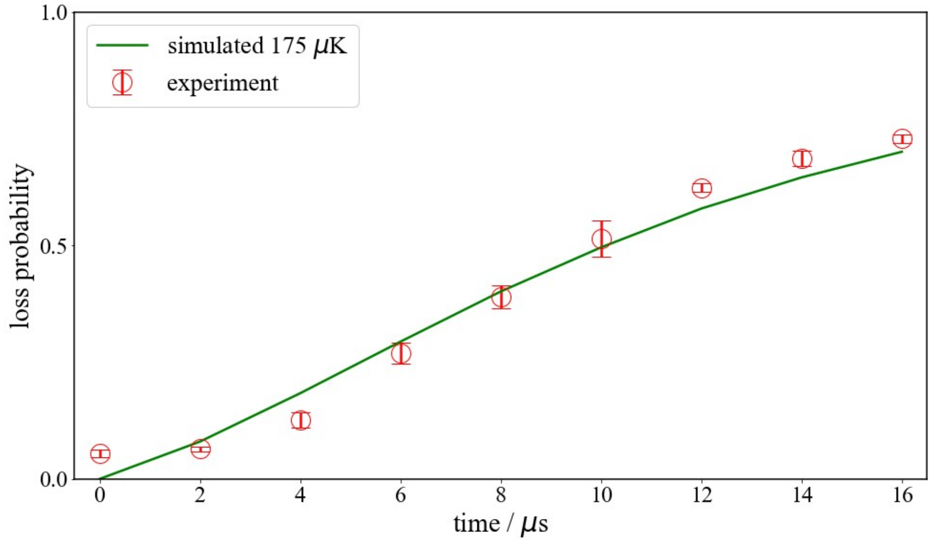

Supplementary Fig. SB1(a)

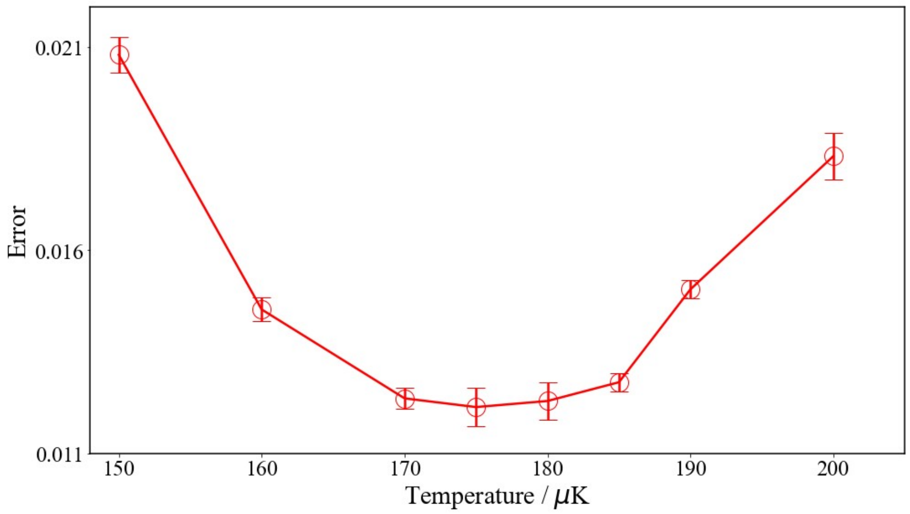

Supplementary Fig. SB1(b)

We agree with the referee that, under these conditions, the initial wavepacket of the single atom indeed populates a set of higher vibrational levels rather than the ground state. Figure 2(b) was intended as a schematic illustration to depict the periodic motion of the atom within the trap and does not imply that the atom is in the ground state. To avoid potential misinterpretation, we have revised Figure 2(b) and its caption accordingly, and clarified this point in the text.

Regarding the influence of the initial temperature on the recapture dynamics, we note that the observed Quantum Zeno Effect (QZE) is most prominent within a moderate temperature range. Based on the loss probability curves, we estimate this range to be approximately 90 μK –200 μK . At lower temperatures, since the intrinsic loss probability of the atom within a single release window is already close to zero, further increasing the measurement frequency leads to additional suppression effects that are difficult to resolve experimentally or numerically, as they are easily obscured by statistical noise. Conversely, at higher temperatures, although our numerical simulations still show a trend of loss suppression with increasing measurement frequency, in the actual experiment, atoms captured directly from the magneto-optical trap (MOT) experience stronger environmental noise and more pronounced system–environment coupling as the atom temperature increases. This decoherence effect (not included in the simplified numerical model used here)

significantly disrupts the system coherence, causing atoms to be lost from the trap with high probability even when nominally confined, so that the measured loss probability after the sequence approaches unity and the QZE becomes difficult to discern.

Concerning thermalization effects, we note that energy-dependent statistical selection always exists during the system evolution, and becomes more pronounced at higher temperatures. However, within the experimental temperature and trap depth range discussed in this work, the atom loss probability exhibits a clear dependence on the measurement frequency, rather than being primarily determined by the trap depth (as shown in Fig. 3, where a threshold exists in the loss probability as a function of trap strength). This pronounced frequency-dependent behavior indicates that the observed loss suppression is not dominated by thermalization or classical energy selection, but arises from measurement-induced dynamical inhibition, i.e., the Quantum Zeno Effect.

Manuscript change:

- Based on the above discussion, we have revised the schematic figures in the manuscript and added clarifications and discussion regarding the initial atom temperature and thermalization effects:
  "It should be further noted that both excessively high and excessively low temperatures hinder the clear observation of the quantum Zeno effect in the experiment. Since the single atoms confined in the optical tweezer are directly loaded from a magneto-optical trap, environmental noise increases with atomic temperature, enhancing the probability of atom loss from the trap. As a result, at higher temperatures, the atomic loss probability after the full experimental sequence approaches unity regardless of the measurement pulse frequency, thereby obscuring the characteristic signatures of the quantum Zeno effect. Conversely, at low temperatures, the intrinsic atom-loss probability within a single release window is already close to zero, making the additional suppression

induced by increasing the measurement frequency difficult to resolve against statistical noise."

3. Given the finite initial temperature (Question 2), all measured data points represent average values across multiple repeated experimental cycles. The authors should clearly state the typical number of repetition times used to obtain each data point and provide a detailed explanation of the statistical methodology employed.

Reply:

In the experiment, each data point is obtained by performing 500 independent realizations of the corresponding experimental sequence. For each parameter setting, the experiment is repeated in three independent batches, from which the statistical mean value and the associated uncertainty are extracted. In the newly added numerical simulations, we directly perform 500 Monte Carlo trajectories to obtain the corresponding averaged quantities.

It should be noted that the loading of a single atom from the magneto-optical trap (MOT) into the optical tweezer is a probabilistic process. As a result, in each experimental batch the number of effective realizations contributing to the statistics is typically around 300, with slight variations between different batches. This number corresponds to the finite probability of successfully loading a single atom from the MOT into the optical tweezer.

Manuscript change:

- We have added a description of the experimental repetition numbers and the statistical analysis procedure in the manuscript.:

  "After completing the full sequence of measurements, the experiment determines whether the atom remains trapped via fluorescence imaging. Correspondingly, in the numerical simulations, the outcome of each stochastic process determines whether the atom ultimately remains in the trap. By repeating the above procedure for a sufficiently large number (N_{surv}) of independent realizations, we

count the number of surviving events N_{surv}, from which the atomic loss probability $P_{loss}=1-N_{surv}/N_{total}$ is extracted. Experimentally, each data set consists of 500 repeated experimental runs, and each data point is obtained by averaging over three independent data sets under identical parameters. Due to the probabilistic loading of single atoms from the MOT, the effective number of realizations per data set is approximately 300. In the Monte Carlo simulations, we directly use 500 trajectories."

4. In Fig. 3, the baseline loss probability without measurement pulses (zero strength I) differs between panels (a), (b), and (c). Please interpret the variation in this zero-strength loss probability across the panels. Does this indicate the atoms were prepared in different initial conditions or temperatures for each experiment? Also, the authors should clarify to what value the measurement pulse strength I is normalized

Reply:

We thank the referee for the careful comment regarding the apparent differences in the baseline loss probability in Fig. 3. As correctly pointed out by the referee, the baseline loss probability at zero measurement strength ($I=0$) appears to differ among Fig. 3(a), (b), and (c). We apologize for this confusion, which originates from an oversight during the plotting process: the experimental data points corresponding to zero measurement strength were inadvertently omitted, leading to an apparent discrepancy in the baselines. In fact, when the measurement pulses are switched off ($I=0$), the loss probability is identical for all three experimental conditions and is close to 1. In these experiments, the initial preparation conditions of the atoms were the same, and the initial temperature was identical for all data sets, measured to be approximately $175\,\mu\mathrm{K}$, as described in response to Question 2. Therefore, the data shown in Fig. 3 do not reflect different initial temperatures or initial state preparation conditions. We have corrected Fig. 3 by adding the missing data points at $I=0$.

Regarding the normalization of the measurement pulse strength I, the measurement strength used in the manuscript is normalized to the optical power of the

tweezer trap. Specifically, $I = 1$ corresponds to an optical power of $8\,\mathrm{mW}$ per tweezer after the objective. In the experiment, the measurement pulse intensity is controlled by adjusting the driving voltage of the acousto-optic modulator (AOM). The calibration curve relating the AOM modulation voltage to the output optical power is provided in Supplementary Fig. SB2.

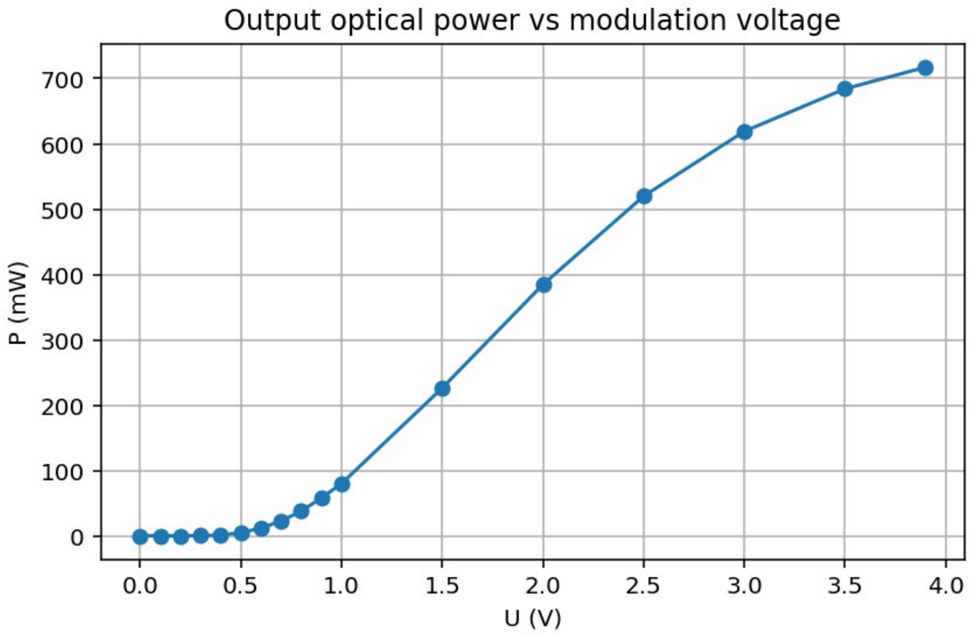

Supplementary Fig. SB2

Manuscript change:

- We have corrected the plotting error and clarified the normalization of the measurement strength in the revised manuscript:

  "The measured atom-loss probabilities as a function of measurement strength are shown in Fig. 3(a－c). The inset of Fig. 3(a) illustrates the experimental time sequence: N measurement pulses of $0.4\ \mu s$ duration are embedded within a $30\ \mu s$ free-evolution window. On this timescale, the unitary evolution induced by the trapping potential is essentially negligible. The variation of the normalized measurement strength I is implemented by adjusting the trap depth, with the reference depth set to $2\ \mathrm{mK}$."

"In the weak-measurement regime, each measurement pulse perturbs the atomic motional state, effectively reducing the outward propagation rate of the wavefunction and thereby slightly suppressing the loss probability compared to free evolution. As the measurement pulse intensity is gradually increased, the influence of a single measurement on the spatial wavefunction becomes progressively stronger. This suppression exhibits a quadratic scaling with measurement strength until I exceeds a threshold value (approximately 0.6), beyond which a single measurement can be well approximated as an ideal projective measurement. In this regime, the recapture probability during an individual measurement is primarily determined by the initial atomic temperature and the evolution time within the release window. Further increasing the measurement strength does not significantly modify the projection induced by each pulse; consequently, the atom-loss probability accumulated over multiple measurements approaches a limiting value determined by the measurement frequency, as indicated by the black dashed line in the figure. Under conditions of frequent strong measurements [Fig 3(c)], the atom-loss probability approaches zero, corresponding to a freezing of the motional dynamics induced by the quantum Zeno effect. These results clearly reveal a crossover from weak-measurement-induced motional perturbations to strong-measurement-dominated wavefunction collapse, and demonstrate that the combined control of measurement strength and measurement frequency provides a flexible and powerful approach for engineering atomic motional quantum states."

5. The loss probability curves in Fig. 3 (a), (b), and (c) reach distinct saturation values (I would call them asymptotic convergence thresholds) when the pulse intensity is increased. The authors should provide a detailed interpretation of this saturation phenomenon in the main text. Additionally, is this asymptotic threshold affected by the heating rate of the 840nm trap? The authors should provide information of the

scattering/heating rate of the 840nm laser. I guess it should introduce relatively neglectable loss probability compared to the recapture effect.

Reply:

We thank the referee for pointing out the saturation behavior of the loss probability curves in Fig. 3(a), (b), and (c) at high measurement pulse intensities, and for raising important questions regarding its physical origin. We agree with the referee's observation that these saturation values can be understood as asymptotic convergence thresholds in the strong-measurement limit, and that they have a clear physical interpretation.

As the measurement pulse intensity is increased, the effect of each individual measurement on the spatial wavefunction of the atom becomes progressively stronger. Once the measurement strength exceeds a certain threshold, a single measurement can be well approximated as an ideal projective measurement. In this regime, the recapture probability in each measurement is primarily determined by the spatial expansion of the atomic wavepacket during the release window and by the temporal structure of the measurement sequence. Further increasing the measurement strength does not significantly modify the projection effect of a single measurement. Consequently, the accumulated loss probability over multiple measurements approaches a limiting value that is determined by the intrinsic system dynamics and the measurement timing, resulting in the saturation behavior observed in Fig. 3.

Furthermore, Fig. 3(a), (b), and (c) correspond to different measurement frequencies (or equivalently, different time intervals between measurements). As a result, even in the strong-measurement limit, the cumulative influence of the measurement sequence on the atomic dynamics differs among the three cases. The distinct asymptotic thresholds observed in the three panels therefore reflect the different dynamical limits associated with different measurement frequencies, as expected.

Regarding whether this asymptotic threshold is affected by heating or scattering induced by the 840 nm optical tweezer, we have performed a corresponding order-of-magnitude estimate. Since the 840 nm tweezer constitutes a highly

far-detuned trap for Rb atoms, the spontaneous scattering rate is strongly suppressed relative to the trap depth. Under the experimental conditions used in this work, the scattering or heating rate is expected to be on the order of a few hertz. Given that both the duration of a single measurement pulse and the total measurement sequence are on the microsecond timescale, the additional heating or loss probability induced by tweezer scattering can be estimated to be on the order of 10^{-4}, which is negligible compared to the loss probability governed by the release–recapture dynamics. We therefore conclude that heating or scattering from the 840 nm tweezer does not play a significant role in determining the saturation thresholds observed in Fig. 3.

Manuscript change:

- We have added a detailed discussion of the physical origin of the saturation behavior in the manuscript and clarified the role of measurement frequency in determining the asymptotic thresholds:

  "Once the measurement pulse duration exceeds this threshold, the loss probability exhibits damped oscillations as a function of the measurement duration. Considering a trap depth of $U_0 = 2$ mK and a beam waist of $\omega_0 = 1.2\ \mu$m, the oscillation period of a ^{87}Rb atom confined in the trap is approximately $\sqrt{m_{\rm Rb}\omega_0^2/(4U_0)} \approx 8.3\ \mu s$, where $m_{\rm Rb}$ denotes the atomic mass. This period is approximately twice the oscillation period observed in the experimental loss probability as a function of τ, in good agreement with the theoretical expectation, indicating that the loss probability faithfully reflects the periodic motion of the atom inside the trap. The damping observed in the data originates from dissipative coupling between the atom and its thermal environment, as well as from higher-order axial modes of the trapping potential, both of which effectively modulate the oscillation amplitude. A sinusoidal function with an exponential envelope provides a good fit to the experimental data. Furthermore, by reducing the measurement pulse intensity by half, we tune the effective trap

frequency. As shown in Fig. 2(d), the Inert Dwell Time ($\sim 2.0\ \mu s$) remains unchanged, while the oscillation period of the loss probability doubles, demonstrating that the back-action of long measurement pulses on the atom is indeed dominated by its unitary evolution inside the trap."

6. The authors should provide information about the trap frequencies. As the trap modulation is performed within several tens of microseconds, should it cause the parametric heating to the single atom? Specifically, is the frequency spectrum resulting from the Fourier transform of the modulation pulses far from or close to the initial trap frequencies?.

Reply:

We thank the referee for the careful question regarding the trap frequency and the possible parametric heating effects.

In our experiment, the optical tweezer trap operates at a wavelength of 840 nm, with a beam waist of approximately $1.2\ \mu\mathrm{m}$ and a trap depth of $2\ \mathrm{mK}$. From these parameters, the motional frequency of a single atom confined in the tweezer trap is estimated to be approximately $120\ \mathrm{kHz}$. This value is physically consistent with the characteristic frequency observed in the experiment for the modulation-period dependence of the loss probability (see Fig. 2(c)), which is approximately twice the trap frequency. Considering that there are two symmetric loss channels within one full oscillation period of the atomic motion, this estimate is in good quantitative agreement with the experimental observation.

The measurement process in the experiment is implemented by square-wave modulation of the optical tweezer trap. Specifically, each measurement cycle consists of a fixed free-evolution interval ($2-9\ \mu s$), followed by a trap-on pulse with a duration of $\tau = 0.5-5\ \mu s$. This sequence is repeated 5-18 times within a single experimental run. As pointed out by the referee, Fourier analysis of the modulation signal reveals that, in some modulation sequences, spectral components indeed

approach the trap frequency or its second harmonic. For example, in the sequence with measurement number N = 15, a total free-evolution time of $3\times15\,\mu s$, and a single trap-on duration of $\tau=5\,\mu s$, the modulation spectrum exhibits a pronounced overlap with the trap frequency (Supplementary Fig. SB3). This indicates that, in principle, parametric heating may occur in certain experimental sequences.

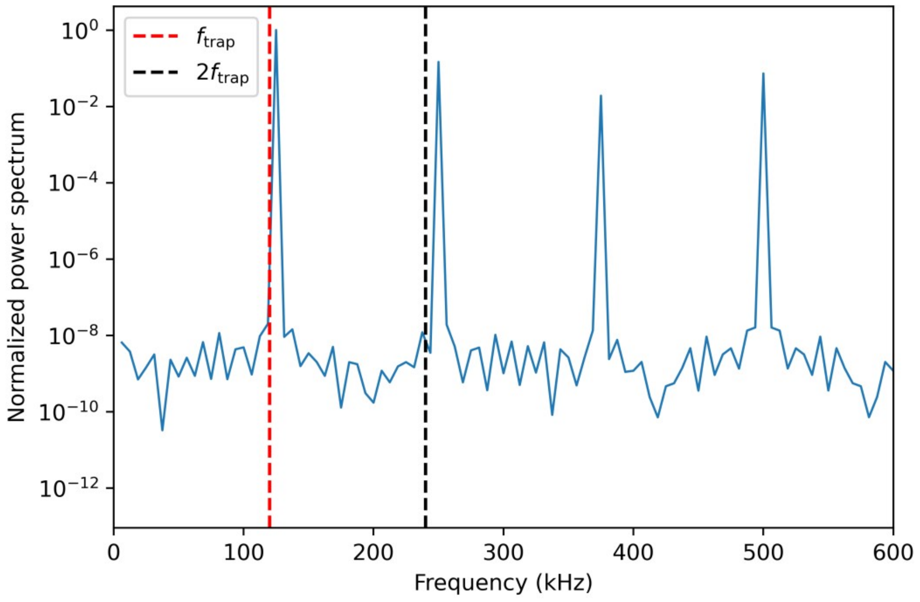

Supplementary Fig. SB3

However, we observe that even in those sequences where the spectral components overlap with the parametric-heating condition, the corresponding atomic loss probability does not show a noticeable increase. More specifically, the overall trend that the loss probability decreases with increasing measurement frequency remains unchanged under these conditions. This indicates that, although parametric heating may introduce an additional loss channel, the observed dynamics are still predominantly governed by measurement-induced dynamical suppression, i.e., the quantum Zeno effect.

In addition, regarding the frequency-shift direction of the modulation spectrum relative to the trap frequency, we have performed a systematic analysis for different experimental parameters. For example, in the sequences corresponding to Fig. 1, when $\tau=0.4\,\mu s$, the spectra for measurement numbers N = 5 and N = 6 show the strongest overlap with the trap frequency and its second harmonic, while further

increasing N leads to a gradual blue shift relative to the trap frequency. In contrast, for $\tau = 5\ \mu s$, the strongest overlap occurs at N = 15, with increasing N resulting in a blue shift and decreasing N leading to a red shift. Similarly, for the sequences shown in Fig. 2(c), the spectrum is closest to the trap frequency at $\tau = 2.2\ \mu s$,with larger τ producing a red shift and smaller τ producing a blue shift. The remaining experimental parameters exhibit analogous behavior: at intermediate parameter values the modulation spectrum may satisfy the parametric-heating condition, while deviations from this region lead to progressive detuning. Nevertheless, as discussed above, even under conditions where spectral overlap occurs, the quantum Zeno effect remains clearly observable and is not masked by possible parametric heating effects.

Manuscript change:

- We have added a clarification of the trap frequency and period in the revised manuscript:

  "Once the measurement pulse duration exceeds this threshold, the loss probability exhibits damped oscillations as a function of the measurement duration. Considering a trap depth of $U_0 = 2$ mK and a beam waist of $\omega_0 = 1.2\ \mu$m, the oscillation period of a ^{87}Rb atom confined in the trap is approximately $\sqrt{m_{\mathrm{Rb}}\omega_0^2/(4U_0)} \approx 8.3\ \mu s$, where m_{Rb} denotes the atomic mass. This period is approximately twice the oscillation period observed in the experimental loss probability as a function of τ, in good agreement with the theoretical expectation, indicating that the loss probability faithfully reflects the periodic motion of the atom inside the trap. The damping observed in the data originates from dissipative coupling between the atom and its thermal environment, as well as from higher-order axial modes of the trapping potential, both of which effectively modulate the oscillation amplitude. A sinusoidal function with an exponential envelope provides a good fit to the experimental data. Furthermore, by reducing the measurement pulse intensity by half, we tune the effective trap

frequency. As shown in Fig. 2(d), the Inert Dwell Time ($\sim 2.0\ \mu s$) remains unchanged, while the oscillation period of the loss probability doubles, demonstrating that the back-action of long measurement pulses on the atom is indeed dominated by its unitary evolution inside the trap."

7. Related to question 6, the modulation of the tweezer trap is very similar to the time-dependent 'TOP' trap in cold atom experiments. That means we can understand that, the single atom feels an effective optical trap in the release window. Can the loss probability be directly related to the effective trap depth/frequencies? Is it possible to unify the data dependencies (on modulation frequency/pulse intensity) using the scale of the effective trap depth/frequency? The survival probability should be related to the 'Franck-Condon' overlap of the initial wave packet with the effective trap's vibrational levels. Can the authors provide brief insights into the feasibility of modeling this process using a fully quantum mechanical language?

Reply:

We thank the reviewer for the constructive suggestion to interpret the experimental protocol in terms of a time-dependent effective trap, and for proposing an intuitive physical picture based on effective trap depth, effective vibrational frequencies, and Franck–Condon overlaps. We agree that this perspective is highly insightful for developing intuition about the atomic motion during the release–recapture dynamics.

Following the reviewer's suggestion, we have supplemented our analysis with a fully quantum-mechanical modeling of the process and performed corresponding Monte Carlo simulations (consistent with our response to Question 1). In this framework, the dynamics explicitly incorporate two distinct physical mechanisms. First, the atomic wave function undergoes unitary time evolution induced by the modulation of the optical tweezer potential. Second, when the trap is switched on, a measurement process occurs that determines whether the atom remains within the bound-state subspace of the trap. In quantum-mechanical terms, this measurement is

described as a non-unitary projection update onto the bound-state subspace. Within this description, the atomic survival probability is defined by the overlap between the atomic quantum state and the trap's bound-state subspace, which can indeed be viewed, at a formal level, as a Franck–Condon–type overlap.

At the same time, we have systematically examined a contrasting scenario in which the dynamics are governed solely by time-dependent unitary evolution, without invoking any measurement process, namely, the trap switching is assumed not to perform a measurement, and the entire evolution is treated as motion in a time-modulated effective potential. By directly comparing this purely unitary model with the measurement-inclusive model, we find that although unitary trap dynamics can influence the atomic evolution and may produce superficially similar suppression trends, several key experimental observations cannot be reproduced without explicitly including the measurement process. Notably, features such as the non-zero loss-probability threshold observed in Fig. 3 and the effective transport rate of moving atoms shown in Fig. 5 emerge only when the measurement-induced non-unitary dynamics are incorporated.

To illustrate this point explicitly, we present in Supplementary Fig. SB4 a direct comparison corresponding to Fig. 3(a) of the main text, where the blue curve represents the model including measurements and the orange curve corresponds to purely unitary evolution. In the purely unitary case, the loss probability decreases monotonically with increasing trap strength and does not exhibit a finite saturation threshold, instead approaching zero asymptotically. This behavior contrasts sharply with the experimental observations. Therefore, within the experimental parameter regime explored in this work, while unitary dynamics induced by the optical tweezer modulation are undoubtedly present, a description based solely on an effective time-averaged trap is insufficient to fully account for the observed behavior.

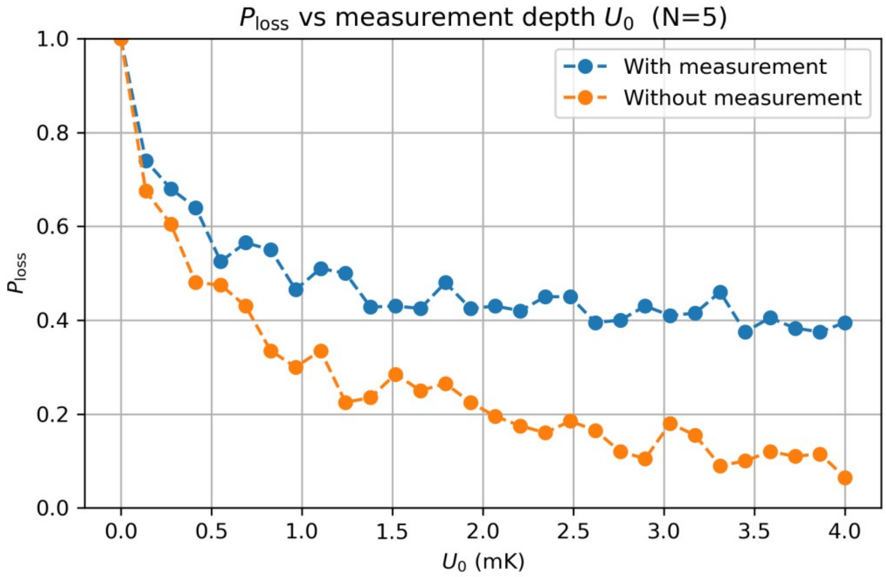

Supplementary Fig. SB4

In summary, both the unitary dynamics induced by trap modulation and the non-unitary dynamics associated with the trap-induced measurement coexist in the experiment and contribute to the system's evolution. However, in the parameter regime relevant to our study, the inclusion of measurement-induced non-unitary dynamics is essential to consistently explain the key experimental observations.

Manuscript change:

- In the modified manuscript, we have described the physical process using a full quantum theory, as shown in the revision for Question 1.

8. Figure 4 shows that the loss is suppressed for short and long pulse durations, but the effect is reduced for the intermediate $3.5 \mu s$ duration. Does this behavior indicate a crossover from the Quantum Zeno to the Anti-Zeno regime? Also, please investigate if the behavior observed at $3.5 \mu s$ relates to the parametric heating discussed in question 6.

Reply:

We thank the referee for the insightful question regarding the reduced suppression effect observed at the intermediate pulse duration ($\tau = 3.5\ \mu s$) in Fig. 4,

and for raising the possibility that this behavior might be related to the anti-Quantum Zeno effect (AZE) or to parametric heating.

We first clarify that the behavior shown in Fig. 4 does not originate from the onset of the anti-Quantum Zeno effect. The AZE typically occurs when the characteristic frequency scale introduced by the measurement process overlaps with the spectral density of the system's decay channels into the environment or other continuum states, thereby enhancing the decay rate. In our experimental system, the escape of the atom from the optical tweezer primarily corresponds to coupling into free-space, i.e., unbound states. The relevant energy scale is set by the difference between the trap depth and the atomic kinetic energy, which corresponds to a frequency scale on the order of tens of MHz for a trap depth of approximately $2\ \mathrm{mK}$. By contrast, the frequency scale associated with the measurement cycle in our experiment lies in the range of tens to a few hundred kHz. For $\tau = 3.5\ \mu s$ in Fig. 4, the corresponding measurement frequency is about 180 kHz, which is far below the dominant spectral weight of the coupling to continuum states. Therefore, from the perspective of spectral overlap, the experimental parameters do not satisfy the necessary conditions for the occurrence of the anti-Quantum Zeno effect.

In addition, as discussed in response to Question 6, we have also examined whether the observed behavior could be attributed to parametric heating. For the intermediate pulse duration $\tau = 3.5\ \mu s$, a single measurement cycle consists of $2\ \mu s$ of free evolution followed by $3.5\ \mu s$ of trap-on time. A Fourier analysis of this modulation sequence shows that its dominant frequency components are located at 180 kHz, 360 kHz, and 540 kHz, none of which are resonant with the trap's fundamental vibrational frequency (approximately 120 kHz) or its primary parametric resonance conditions. Consequently, the reduced suppression observed at this intermediate pulse duration cannot be attributed to parametric heating.

Based on these considerations, we interpret the relatively weaker suppression at $\tau = 3.5\ \mu s$ in Fig. 4 as arising from a phase-matching effect between the trap-induced

coherent dynamics and the release timing. Specifically, this pulse duration corresponds to release times that are close to particular phases of the atomic motion inside the trap, at which the atomic wave packet is more likely to exceed the effective capture condition of the trap in either position or momentum space, resulting in an increased loss probability during a single release event. This interpretation is consistent with the periodic behavior observed in Fig. 2, where the loss probability is maximal around $\tau = 3.5\ \mu s$. The effect therefore reflects the matching between the intra-trap coherent dynamics and the temporal structure of the release process, rather than an enhancement of decay channels induced by the measurement frequency.

Manuscript change:

- We have added a more detailed discussion of this phenomenon in the revised manuscript:

  "When the goal is to suppress the atomic motion, frequent short measurement pulses ($\tau = 1\ \mu s$) strongly localize the atomic wave packet near the center of the trapping potential, allowing it to remain in a low-loss motional state even during long intervals of free evolution [Fig. 4(b), red data points]. In contrast, when the objective is to facilitate the escape of atoms from the trap, we apply measurement pulses with a duration matched to one quarter of the trap oscillation period, $\tau = 3.5\ \mu s$. In this regime, the unitary evolution inside the trap induces an effective phase inversion of wavefunction components that would otherwise favor confinement, thereby degrading their spatial localization during the subsequent free evolution and significantly reducing the probability of atomic recapture [Fig. 4(b), blue data points]."

  "Notably, although thermal noise introduces additional loss channels for atoms inside the trap, the atomic loss probability does not vary monotonically with the measurement pulse duration. Specifically, when the pulse duration is extended to half of the trap oscillation period, $\tau = 5.0\ \mu s$, the oscillatory dynamics of the trap

partially restore the spatial localization of the wave packet, leading to a reduced loss probability during the release process. Experimentally, this manifests as the reemergence of low-loss behavior at specific evolution times, as indicated by the green data points in Fig. 4(b). These results clearly demonstrate that measurements can be employed not only to suppress or enhance atomic motion but also, when properly engineered, to serve as a versatile resource for tailoring and controlling atomic motional states, enabling effective manipulation of atomic dynamics."

9. In Fig. 5, the authors show how to control the atomic motion by modulating the trap parameters. Are there any advantages for such pulsed modulation compared to the case by just directly ramping the driving frequency of the AOD adiabatically? The authors claim this method avoids additional momentum transfer compared to adiabatically ramping the AOD driving frequency. So, to support this claim, the authors should measure and show the temperature of the single atom after the directional moving in Fig. 5, comparing it to the initial value. Also, a valuable addition for future applications would be a detailed discussion of the possible limitations of this transport scheme, e.g., maximum moving speed, coherence lifetime, and heating effects.

Reply:

We thank the reviewer for the insightful questions regarding the directed atomic transport scheme shown in Fig. 5, as well as for the constructive suggestion to compare this approach with the conventional trap-dragging method based on adiabatic scanning of the AOD driving frequency.

In our experimental scheme, the spatial transport of the atom does not arise from continuous dragging by the trapping potential. Instead, it originates from the quantum measurement backaction induced by the spatial displacement of the optical tweezer trap during each measurement cycle. Compared with the dragging scheme based on adiabatic AOD frequency ramps, a key feature of our method is that the effective atomic transport velocity is no longer constrained by adiabaticity requirements. As

shown in the manuscript, under the present experimental parameters, we achieve an effective atomic drift velocity of approximately $0.7\,m/s$, which is significantly higher than the maximum stable velocity attainable via trap dragging in the same experimental system (about $0.05\,m/s$). This enhancement in controllable transport speed may enable atomic manipulation over larger spatial scales within limited experimental time, for example, facilitating large-scale rearrangement in atomic arrays.

Regarding the statement that this method “avoids imparting additional momentum,” we would like to further clarify its intended meaning. This statement is primarily based on the physical picture of the measurement process: in this scheme, the directed atomic motion can be understood as a gradual shift of the spatial position at which the measurement operator acts (or, equivalently, a translation of the measurement’s spatial distribution), rather than the application of a directional force by the trapping potential. Within this idealized picture, the atomic wavefunction does not acquire an additional deterministic momentum component during the projection process. However, under realistic experimental conditions, several effects, including phase evolution induced by changes in the trap position, atomic dynamics during the finite trap-on time, and possible parametric heating associated with the experimental sequence—may introduce additional heating. As correctly pointed out by the reviewer, directly measuring the atomic temperature before and after the directed transport would provide a more quantitative assessment of possible heating. Together with the potential limitations discussed below, this constitutes an important direction for future work.

Concerning the potential limitations and applicability of this transport scheme, we identify several key aspects. First, although measurement-induced transport can exceed the maximum velocity allowed by adiabatic trap dragging, in the low-velocity regime where adiabatic dragging is already applicable, the measurement-induced scheme may exhibit a higher atom-loss probability. This is because the trap must be periodically switched off during the measurement sequence, allowing the atomic

wavefunction to expand in free space, whereas in adiabatic dragging the atom remains continuously confined. Second, the maximum achievable transport rate in the measurement-induced scheme is limited by the atomic temperature: higher temperatures lead to faster wavefunction expansion during free evolution, requiring smaller spatial separations between consecutive measurement positions for a given time interval in order to maintain sufficient overlap with the bound state of the trap; otherwise, the measurement process is more likely to result in atom loss. Finally, since the trap is necessarily switched on for a finite duration after each projection, the subsequent trap-induced coherent dynamics may, under certain parameter conditions (e.g., specific phase-matching scenarios), introduce additional heating effects. As the primary goal of this work is to demonstrate the new physical mechanism of measurement-induced directed transport and its experimental feasibility, a detailed quantitative investigation of these issues is left as a central objective of future studies.

Manuscript change:

- We have added corresponding discussions in the revised manuscript to more clearly elucidate the physical mechanism of this scheme, as well as its advantages and potential limitations:

  "Under such a sequence, the role of frequent measurements is no longer to freeze the atom at a fixed position. Instead, the atomic wavefunction is repeatedly projected in a directional manner, with the wave packet center being transferred step by step to the position of the dipole trap, i.e., measurement-induced directed motion of the atom, as illustrated in Fig. 5(b). Experimentally, we first perform in-situ imaging of the atom (Fig. 5(c1)), followed by global imaging for measurement numbers $\mathrm{N}=20$ (Fig. 5(c2)) and $\mathrm{N}=40$ (Fig. 5(c3)). The results clearly show the atom bound in the dipole trap displaced by approximately $2\ \mu\mathrm{m}$ and $4\ \mu\mathrm{m}$ from the initial position, respectively. It is worth emphasizing that, in this process, the effective drift velocity of the atom is approximately $0.7\ \mathrm{m/s}$, which is significantly higher than the maximum velocity of $0.05\ \mathrm{m/s}$

achievable through trap dragging with the optical tweezer kept continuously on in the same experimental system. This result demonstrates that measurement-induced directed atomic motion is no longer constrained by the adiabatic condition associated with unitary dynamics, thereby providing a new paradigm for controlling atomic displacement.”

“From a fundamental perspective, however, the maximum transport rate achievable with this measurement-induced scheme is still limited by the atomic temperature. Higher temperatures lead to more rapid expansion of the atomic wavefunction during free evolution, and thus, for a given time interval, require a corresponding reduction in the spatial separation between successive measurement positions in order to maintain sufficient overlap with the bound states of the trap. Otherwise, atom loss induced during the measurement process can substantially reduce the efficiency of atomic control. Consequently, this approach is particularly well suited for applications involving low-temperature atoms and scenarios in which a certain degree of tolerance to single-step loss is acceptable.”

10. Minor comments:

(1) In the introductory discussion of QZE experimental observations (first page), please reference key experiments from other cold atom platforms, such as those involving ultracold molecules [Phys. Rev. Lett. 112, 070404 (2014)], momentum space lattices [npj Quantum Information 7, 78 (2021)], quantum gases [Nat. Commun. 10, 855 (2019), Science 349, 1317 (2015)], and Rydberg atoms [Nat. Phys. 10, 715 (2014)].

(2) I suggest adding a dedicated subsection in the Method section providing more quantitative details about the data analysis, systematic uncertainties, and calibration procedures.

(3) Phrasing check: The phrasing, such as "enabling deterministic preparation of distinct motional states," should be carefully clarified. The word "deterministic" seems ambiguous given the current evidence and may be an overstatement. A careful

proofread for typos and minor language issues throughout the manuscript is recommended.

Reply:

We thank the referee for these comments and have revised the manuscript accordingly by incorporating the suggested references, adding more detailed quantitative analyses and experimental details in the main text, and carefully revising the phrasing and language throughout the manuscript.

REVIEWER COMMENTS

Reviewer #1:

The revised version has satisfactorily addressed the outstanding technical issues. However, my main objection has remained unanswered. The protocol describes a sequence of unitary operations caused by alternating Hamiltonians but not projective measurements. Thus the connection to the QZE is inappropriate.

A secondary remark is that if this were the QZE, then there should be also the AZE for other parameters. However, this does not seem to be the case. The paper cannot be published on the basis of the material and interpretation at hand. I can only reiterate that the paper is conceptually flawed and does not describe the quantum Zeno effect. The same objections remain as in my first report and the new version does not address them.

Reply:

We thank the reviewer for the careful reading of our manuscript and for reiterating the central concern regarding the interpretation of the dynamics.

We would like to clarify that, although the experimental sequence involves alternating Hamiltonians, the switching of the optical tweezer does not correspond to purely unitary evolution. Instead, each trap-on event intrinsically involves a measurement process that determines whether the atomic wavefunction remains within the bound-state subspace of the trap. Physically, this corresponds to a recapture-versus-loss process: the component of the wavefunction overlapping with the bound states continues the evolution, while the orthogonal component irreversibly couples to continuum states, leading to atom loss. This loss channel constitutes a genuine measurement backaction and introduces essential non-unitarity into the dynamics.

Crucially, a purely unitary "stop-and-go" description is inconsistent with key experimental observations. In particular, (i) the non-zero saturation of the loss probability at large trap depth (Fig. 3), and (ii) the effective transport velocity exceeding the adiabatic dragging limit (Fig. 5), cannot be reproduced within any fully unitary framework. We have explicitly verified this by comparing models with and without

measurement backaction in previous reply, where only the measurement-inclusive description is consistent with the experimental data. These results provide direct evidence that the observed dynamics cannot be understood as purely unitary evolution.

Regarding the absence of the anti-Zeno effect, we note that within the parameter regime explored in this experiment, the relevant measurement frequency remains far below the characteristic energy scale associated with coupling to continuum states. As a result, the conditions required for the anti-Zeno effect are not satisfied, and its absence is therefore expected and does not contradict the presence of measurement-induced Zeno dynamics.

In light of these clarifications, we respectfully maintain that the experimental observations are governed by measurement-induced suppression of dynamics and are appropriately interpreted within the framework of quantum Zeno-type behavior.

==

REVIEWER COMMENTS

Reviewer #2:

The authors address all related questions properly, and now I recommend publication.

Reply:

We thank the reviewer for the positive assessment of our work and for the constructive and valuable comments, which have significantly helped improve the quality and clarity of the manuscript.
